# Anti-hemagglutinin monomeric nanobody provides prophylactic immunity against H1 subtype influenza A viruses

**DOI:** 10.1371/journal.pone.0301664

**Published:** 2024-07-10

**Authors:** Elena Susana Barbieri, Carla Sosa-Holt, Lorena Itati Ibañez, Josefina Baztarrica, Lorena Garaicoechea, Claire Lindsey Gay, Carlos Joaquin Caceres, Matias Aduriz, Elsa Baumeister, José Angel Escribano, Daniel Perez, Andrés Wigdorovitz, Gladys Viviana Parreño, Mariana Puntel

**Affiliations:** 1 Virology Institute IncuINTA (IVIT-CONICET), National Institute of Agricultural Technology, Hurlingham, Buenos Aires, Argentina; 2 National Council for Scientific and Technical Research (CONICET), Buenos Aires City, Buenos Aires, Argentina; 3 Institute of Science and Technology, Buenos Aires City, Buenos Aires, Argentina; 4 Department of Population Health, College of Veterinary Medicine, University of Georgia, Athens, Georgia, United States of America; 5 National Institute of Infectious Diseases, Malbran Institute, Buenos Aires City, Buenos Aires, Argentina; 6 Algenex S.L., Madrid, Spain; Instituto Butantan, BRAZIL

## Abstract

Influenza viruses constitute a major threat to human health globally. The viral surface glycoprotein hemagglutinin (HA) is the immunodominant antigen, contains the site for binding to the cellular receptor (RBS), and it is the major target of neutralizing antibody responses post-infection. We developed llama-derived single chain antibody fragments (VHHs) specific for type A influenza virus. Four VHHs were identified and further characterized. VHH D81 bound residues in the proximity of the C-terminal region of HA1 of H1 and H5 subtypes, and showed weak neutralizing activity, whereas VHH B33 bound residues in the proximity of the N-terminal region of the HA’s stem domain (HA2) of H1, H5, and H9 subtypes, and showed no neutralizing activity. Of most relevance, VHHs E13 and G41 recognized highly conserved conformational epitopes on the H1 HA’s globular domain (HA1) and showed high virus neutralizing activity (ranging between 0.94 to 0.01μM), when tested against several human H1N1 isolates. Additionally, E13 displayed abrogated virus replication of a panel of H1N1 strains spanning over 80 years of antigenic drift and isolated from human, avian, and swine origin. Interestingly, E13 conferred protection in vivo at a dose as low as 0.05 mg/kg. Mice treated with E13 intranasally resulted in undetectable virus challenge loads in the lungs at day 4 post-challenge. The transfer of sterilizing pan-H1 immunity, by a dose in the range of micrograms given intranasally, is of major significance for a monomeric VHH and supports the further development of E13 as an immunotherapeutic agent for the mitigation of influenza infections.

## Introduction

Influenza A viruses (FLUAVs) are a continuous threat to the health of humans and animals. FLUAVs are enveloped viruses in the family Orthomyxoviridae sharing negative sense single stranded RNA genomes (-ssRNA). FLUAVs contain 8 -ssRNAs that encode 10 major open reading frames and additional minor open reading frames whose expression and role in infection is largely strain dependent. FLUAVs encode two viral surface glycoproteins, hemagglutinin (HA) and neuraminidase (NA). The antigenic properties of these two proteins allow the subdivision of FLUAVs into H and N subtypes. FLUAVs in many subtype combinations have been described, particularly in wild aquatic birds of the orders Anseriformes and Charadriiformes, which are considered the natural hosts of 16 HA (H1-H16) and 9 NA (N1-N9) subtypes [1]. From this primordial reservoir, FLUAVs occasionally emerge causing outbreaks in poultry and mammals and where viruses have an opportunity to further adapt and become established. It is commonly accepted that non-natural hosts of FLUAVs act as intermediaries hosts in the emergence of zoonotic and pandemic strains. Through time, humans have experienced multiple pandemic episodes associated to the emergence of new FLUAV strains. The pandemics of 1918 (H1N1), 1957 (H2N2), 1968 (H3N2), and 2009 (H1N1) highlight the significant toll that FLUAVs have had on human health [2]. Once in humans, pandemic strains eventually become further adapted and associated with seasonal influenza epidemics having the greatest impact on the elderly and very young populations. Seasonal FLUAVs circulating currently in humans are descendants of the 1968 (H3N2) and the 2009 (H1N1) strains [3, 4].

Vaccines are the best tool to prevent influenza. The HA is major target of neutralizing responses against FLUAVs and thus the major target in vaccine formulations. However, the error-prone nature of FLUAVs and their ability to tolerate mutations without severely affecting virus fitness allows the virus to evade prior immune responses. Thus, vaccines are reformulated periodically, and people require re-vaccinations. Although approved antivirals are available for the treatment of severe cases of influenza, resistant strains can emerge readily making such treatment obsolete [3].

The HA projects from the virus surface as a ~220kD homotrimer stabilized by coiled-coil interactions and anchored on the virus envelope by transmembrane domains in each monomer [5]. Each monomer contains two subunits linked by di-sulfide bonds, the globular head (HA1) and the stalk, formed by HA2 plus the N- and the C-termini domains of the HA1 subunit [6]. The HA mediates viral entry into the host cell by attaching to N-acetylneuraminic acid (sialic acid) present on glycoconjugates on the surface of the host cell. Such binding occurs via a shallow pocket known as the receptor-binding site (RBS) located on the top of the globular head of HA [7]. The RBS is characterized by four elements: the 130-loop, the 190-alpha helix, the 220-loop, and a hydrogen-bonded network of conserved amino acids at positions 98, 153, 183, and 195 (H3 HA numbering) [8]. In the H1 HA, the antigenic relevant sites are distributed roughly over five conformational epitopes on the globular head of the distal tip on each monomer (Sa and Sb sites) and proximal to the stalk region (Ca1, Ca2 and Cb). The RBS is located between the Sa, Sb and Ca2 regions. The majority of the epitopes on HA contain highly variable, protruding loops that can be accessed readily by approaching antibodies [9]. Passive immunotherapy based on the administration of specific antibodies targeted directly against a desired antigen, has been shown to significantly reduce the mortality and mechanical ventilation requirements of patients with virus-induced severe acute respiratory infections [10]. However, the amino acid sequence diversity in regions targeted by conventional antibodies limit the spectrum of protection that they could provide.

Recombinant nanobodies from *Camelidae* species derived from the variable domain of heavy chain antibodies (VHHs) constitute a promising alternative to the conventional immunoglobulins. The single polypeptide that constitutes the VHH possesses low molecular weight (~15 kDa) while retaining full antigen binding capacity. Unlike single-chain variable fragment (scFv) antibodies, the conformation of the paratope of VHHs does not require the VL fragment typically found in immunoglobulins [11]. Additional advantages of VHHs include stability, solubility, and ease of preparation of cDNA libraries for the selection of those with high antigen binding capacity [12]. Promising VHHs with anti-FLUAV activity and therapeutic potential have been previously described [13–15]. Herein we took advantage of the nanobodies features as a way to develop highly specific HA binders with therapeutic potential for the control of influenza infections. We have isolated two sets of nanobodies, including heterosubtypic influenza binders with low or none neutralizing activity on one hand, and pan-H1 neutralizers on the other hand. For that purpose, we developed a VHH library by phage display derived from an immune llama and selected with H1 recombinant HA. The designated candidates were characterized for their binding specificity against H1, H5, H9 and H3 virus subtypes. The neutralizing activity against H1 and H3 viruses from human and animal origin was initially explored. For the most potent neutralizers, we extended the neutralization analysis to H1 isolates both human and animal sources, spanning more than 80 years of viral evolution. For the best candidates on each category, we investigated the location of their antibody epitopes as well as their docking interactions, in silico. Finally, we assessed the therapeutical potential for the neutralizing VHHs, and explored the effective dose for the best candidates, in the mouse model. Together, our findings support H1-specific VHHs as strong candidates for the local prophylaxis of H1 FLUAV infections.

## Materials and methods

### Viruses and cells

A/PuertoRico/8/1934 (H1N1) (hu/PR8/34) virus, and a low pathogenicity avian influenza virus (LPAIV) H5N1 (hu/Viet/04) is a reverse genetics-derived reassortant of HA and NA of A/Vietnam/1194/2004 (H5N1) in the background of A/PR/8/34 (H1N1) virus, where the HA- and neuraminidase coding segments of NIBRG-14 are from A/Vietnam/1194/2004, where the HA segment lacks the polybasic cleavage site, both kindly provided by Dr. Xavier Saelens (VIB, University of Ghent, Belgium) [13]. A/Argentina/017/2009pdm (H1N1) (hu/Arg/09) and A/Perth/16/2009 (H3N2) (hu/Perth/09), were kindly provided by Dr. E. Baumeister (National Institute of Infectious Diseases, Malbran Institute, Argentina) [16]. The mouse adapted A/Argentina/017/2009pdm (hu/Arg/09 ma) was obtained as described in S5 Fig.

The A/Brisbane/59/07(H1N1) (hu/Bri/07) virus, mouse adapted A/California/04/09 (H1N1) virus (hu/Ca/09 ma) [17], a recombinant hu/Ca/09 ma virus carrying a chimeric PB1 gene with Nano luciferase (NLuc) downstream the PB1 open reading frame (hu/Ca/09 ma NLuc) [18], A/Ruddy Turnstone/Delaware/300/09 (H1N1) (rt/Del/09), swine A/swine/South Dakota/2018 (H1N1) (sw/SD/18), and A/turkey/Ohio/313053/2004 (H3N2) virus (ty/Oh/04 NLuc) [18], were kindly provided by Dr. Daniel Perez (University of Georgia, Athens, USA). The H9N1 (gf/HK/99) is the product of reverse genetics reassortant of the A/guinea fowl/Hong Kong/WF10/1999 (H9N2) HA from D. Perez laboratory, in the background of A/PR8/34 (H1N1) [19] (S1 Table).

All viruses were propagated in Madin-Darby Canine Kidney (MDCK) cells or 10-day old embryonated chicken eggs. MDCK cells were grown in Dulbecco´s modified Minimal Essential Medium (D-MEM), supplemented with 10% fetal bovine serum (FBS) (InterNegocios, Argentina), L-1-tosylamide-2-phenylethyl chloro-methyl ketone (TPCK)-treated trypsin (Sigma-Aldrich, U.S.) (TPCK) treated trypsin (2ug/ml), and penicillin-streptomycin 100 U/ml -100 μg/ml (Invitrogen, U.S.). Viral infections were performed as described elsewhere [20]. Virus stocks were harvested after full cytopathic effect was observed and stored at -80°C until further use. TCID_50_ titer were determined using the Reed & Muench method [21].

### Llama VHH development

The study was approved by the Institutional Animal Care and Use Committee (IACUC) of the Instituto Nacional de Tecnologia Agropecuaria, Castelar (INTA-Castelar, protocol # 08–11). A single one-year-old male llama provided from a private breeder was transported to the specialized llama facility located at INTA, following regulations of the National Service of Health and Food Quality (SENASA). The llama was housed in a paddock specially prepared for the VHH line of research as described previously [22]. Llama management, inoculation, and blood sample collection were conducted by trained personnel under the supervision of a veterinarian in accordance with the national and international guidelines for animal welfare. Prior to vaccination the animal was evaluated and confirmed to be seronegative for the presence of influenza-specific antibodies. The llama received a single 3 ml dose of clostridial polyvalent vaccine, (CDVac Clostrimax T) and then immunized by intramuscular injection (i.m.) with 4 doses of monovalent influenza A/California/07/2009 (H1N1) vaccine (Novartis®) on days 0, 15, 165, and 193. On days 347 and 383, the llama received an additional dose of the monovalent influenza A/California/07/2009 (H1N1) and a dose of the trivalent A/California/07/2009 (H1N1), A/Perth/16/2009 (H3N2) and B/Brisbane/60/2008 vaccine (Fluvirin®). The first immunization of the scheme was adjuvated with complete Freund’s adjuvant and the following immunization with incomplete Freund’s adjuvant (1:1 ratio, in a 4.5 ml final volume). The monovalent and trivalent vaccines were injected separately at different immunization sites. No adverse effects were observed immediately after vaccine administration or at any time point post-vaccination. On day 387 post-vaccination, 450 mls of blood were collected by puncture of the jugular vein. After the bleeding step, the llama remained in the paddock for 6 more months in order to confirm the absence of adverse effects due to the immunization and blood sampling, and it was subsequently sent back to the farm of origin.

### VHH library production

The blood sample collected 4 days after the last immunization and the total of mononuclear cells (5.3x 10^8^ cells) obtained was processed for total RNA extraction, cDNA synthesis, and nested PCR amplification. The resulting amplicons were ligated to the phagemid vector pHEN4 as described previously [23].

### Enrichment in VHH of interest

Enrichment for specific binders was performed by the phage display technology applying three rounds of *in vitro* selection. The bacteria containing the VHH library were infected with M13K07 helper phage (Life Technologies, U.S.), and phage particles expressing the VHH repertoire were rescued and precipitated with polyethylene glycol as described previously [24], using recombinant proteins. Preparations of HA0 of A/Puerto Rico/8/1934 (H1N1) virus, and the matrix protein 2 ectodomain for influenza A (M2e), consisting of four tandem copies of the M2e peptide fused to the 4x histidine [(MSLLTEVETPIRNEWGCRCNGSSD)x4 His], were kindly provided by Dr. Escribano (Algenex, Spain) [25] Ninety-six flat-bottom well Maxisorp ELISA plates (NUNC Thermo Scientific) were coated overnight (ON) at 4°C with 0.5 μg/well of either recombinant proteins HA0 or M2e A non-relevant protein (non-infected MDCK cell lysate) and/or PBS were used as negative controls. After blocking, phages were added to the plates. Bound phages were eluted with 100 mM triethylamine (pH 10.0) and immediately neutralized with 1 M Tris-HCl (pH 7.4). The eluted phages were used to infect exponentially growing *E*. *coli* TG1 cells for a second round of panning [23]. Individual colonies were grown and the VHH clones were analyzed by phage ELISA.

### Screening of H1N1-specific VHH clones by phage ELISA

Phages displaying the selected VHHs were produced from individual TG1 clones as previously described [23]. Ninety-six flat-bottom well Maxisorp ELISA plates (NUNC Thermo Scientific, U.S.) were coated with either recombinant HA0, M2e, or PBS. After a blocking step, phages were added and incubated at 37°C for 1 h. Plates were washed with PBST and 50 μl/well of horseradish peroxidase conjugated monoclonal antibody anti-M13 p8 (GE Healthcare, U.S.) was added to detect specific phage binding. After incubation, the plates were washed with PBST and the assay was developed with H_2_O_2_/2,2’-Azinobis [3-ethylbenzothiazoline-6-sulfonic acid]-di ammonium salt (ABTS, KPL, U.S) as substrate/chromogen system, added at 100 μl/well. The OD at 405 nm was measured by an ELISA reader (Multiskan EX, Thermo Scientific,U.S.). Sera from immunized and non-immunized llama and rabbit were included as positive and negative controls, respectively. Clones with the highest positive signal in phage ELISA were selected for further studies.

### VHH colony PCR and sequence analysis

Individual colonies were added to each PCR mix and amplified using Universal reverse primer (5’-TCACACAGGAAACAGCTATGAC-3’) and GIII primer (5’-CCACAGACAGCCCTCATAG-3’) [26]. Taq polymerase (Invitrogen, Brazil) was used according to the manufacturer’s recommendations. Clone diversity was analyzed by Hinf I (Promega, U.S.) enzymatic digestion of the PCR product followed by electrophoresis using a 2% agarose gel. DNA sequencing was performed at the Sequencing Facility (Biotechnology Institute, INTA-Castelar) using purified miniprep DNA QIAprep Spin Miniprep (Qiagen) and M13Rev oligonucleotide. All sequences were aligned by ClustalW with Mega 6.06 and the alignment was edited using BioEdit 7.2.

### Expression and purification of recombinant VHH

The procedure was performed as previously described [23]. Briefly, cDNA of VHH clones in pHEN4 were sub-cloned into the expression vector pHEN6 using Sfi I and Not I restriction enzymes [27]. *E*. *coli* XL1-Blue cells were freshly transformed with the different plasmid constructs. The production of recombinant monovalent VHH was carried out by growing transformed XL1-Blue cells in liquid cultures using Terrific Broth supplemented with ampicillin at 37°C in a shaker at 250 rpm. VHH expression was then induced with 1 mM IPTG (isopropyl-D-thiogalactopyranoside), for 16 h at 28°C. The cells were pelleted and the periplasmic proteins were extracted by osmotic shock [22]. The VHHs were purified from this periplasmic extract by using a High-Trap HP Ni-chelating column (GE Healthcare, U.S.). The integrity of the purified molecules was visualized by 15% sodium dodecyl sulfate-polyacrylamide gel electrophoresis (SDS-PAGE) after Coomassie Brilliant Blue R-250 staining. The endotoxin content of the purified VHHs was 1< EU/μg of protein, as determined by the limulus amebocyte lysate test (GenScript, NJ, U.S.) according to the manufacturer’s instructions [28].

### H1N1 rabbit and mouse serum

Rabbit immune serum was obtained by the immunization service of the animal facility at the Faculty of Exact and Natural Sciences (FCE y N, UBA, Argentina), after three sequential intramuscular immunizations (days 0, 15 and 21) of 0.5 ml/dose of the same commercial trivalent vaccine (Novartis®) containing A/California/07/2009 (H1N1), A/Perth/16/2009 (H3N2) and B/Brisbane/60/2008, used for the llama immunization. Negative control serum was collected from the same animal before starting the immunization scheme. Immune mouse serum (α-H1N1) was obtained after subcutaneous administration of one dose of the experimental vaccine described above, formulated with incomplete Freund´s adjuvant. A dose of 100 μl was given to 8-week-old female BALB/c (n = 14). Final bleeding was performed 21 days after immunization. Antibody level was analyzed for each sample individually by HA0 ELISA and H1N1 ELISA, samples with ELISA titers greater than 1:2,048 were pooled. Blood samples collected after final bleeding were processed for the obtention of serum.

### Virus and protein ELISA

Antibody titers in immune serum from llama, rabbit, and mouse; as well as specificity of the VHH fragments were determined by ELISA assay as described elsewhere [25]. Briefly, 0.5 μg/100ul of the recombinant protein HA0 or M2e were used for coating. Rabbit anti-VHH serum was added to each well, and the bound antibodies were detected with a goat anti-rabbit IgG horseradish peroxidase (HRP)–conjugated (KPL, U.S.). Llama IgG was detected with a goat anti-llama IgG HRP conjugated (Bethyl, U.S.) and mouse IgG was detected with goat anti-mouse IgG HRP conjugated (Bethyl, U.S.). The assay was developed with H_2_O_2_/ABTS. The absorbance measured at 405 nm (OD_405_). The cut off was defined as twice the absorbance obtained in the blank wells.

Specific reactivity was analyzed by ELISA using 16 HU of either influenza virus, or MDCK cell supernatant as a negative control. After blocking, fivefold serially diluted VHHs, starting from a concentration of 2 μM, were tested. Bound VHHs were detected by HRP-conjugated anti-6xHis tag antibody. The assay was developed with 3,3’,5,5’-tetramethylbenzidine (TMB) substrate (Lifetech, U.S.) and absorbance was measured at 450 nm. Medium effective concentration (EC_50_) defined as the concentration of VHH required to reach 50% of the specific binding. EC_50_ was determined after plotting the logarithm of the molar concentration for each nanobody versus the absorbance reading at 450nm. EC_50_ values were calculated using sigmoidal dose response analysis of non-linear data, using GraphPad Prism 5. EC_50_ values >1000nM were considered out of the range of detection of the assay.

To remove the globular HA1 subunit the acid treatment followed by reducing buffer was performed. After coating with virus or MDCK cell supernatant, plates (Greiner, microlon, U.S.) were blocked. Acid treatment was performed with pH 5.5 buffered solution (15 mM citric acid and 150 mM NaCl), treated with 0.1 M dithiothreitol (DTT) to reduce the disulfide bond that connects HA1 to HA2 [25]. VHHs were analyzed in 5-fold dilutions, starting at 40 μg/well. Bound VHHs were detected by the addition of anti-6x histidine-HRP, and developed by TMB and stopped with 1 M H_2_SO_4_. The plates were read at 450 nm. Positive binding was defined as the reciprocal of the highest dilution with an optical density at 450 nm (OD450) that at least doubles the reading of the negative controls.

For the analysis of linear epitopes, a peptide array derived from A/California/04/2009 (H1N1) pdm09 HA (NR-15433, BEI Resources, U.S.) was used. For that purpose, coating was performed individually adding each of 139 peptides (4 ng/well) individually, to a 96 well plate (NUNC Thermo scientific, U.S.). As positive coating control, hu/PR8/34 viral antigen was included. After blocking, a concentration of 20 ng/well of VHH was tested, revealed using a rabbit anti-VHH serum followed by anti-rabbit IgG horseradish peroxidase (HRP)–conjugated (KPL, U.S.), developed with 3,3’,5,5’-tetramethylbenzidine (TMB) substrate (Lifetech, U.S.), and absorbance was read at 450 nm.

### Hemagglutination inhibition assay (HI)

This assay was performed according to the WHO standard protocols [29]. Briefly, two-fold serial dilutions of each VHH (range from 0.5 μg to 0.010 μg) was tested against 8 hemagglutinating units (HU) of virus using freshly prepared 0.75% suspension of guinea pig red blood cells. The HI titer of each VHH was defined as the lowest antibody concentration that completely prevented hemagglutination. Serum of hyperimmunized and non-immunized llama were included as positive and negative controls, respectively.

### Microneutralization assay

Neutralizing VHH titers were determined by microneutralization (MN) assays performed on MDCK cells following the procedure described by WHO [30] with some modifications. Briefly, two-fold serial dilutions of VHH samples (range from 12.5 μg to 7.5 ng) were individually tested against 100 TCID_50_ of the virus, on MDCK cells monolayers. The presence of viral infection was detected by cell ELISA as described elsewhere [25]. Neutralizing Ab titer was expressed as the inverse of the highest dilution which inhibits 50% of the viral infection. The cut off value was calculated subtracting the average absorbance obtained from control cells.

For the neutralizing efficacy against nanoluciferase (NLuc) expressing viruses, luciferase activity was performed as described elsewhere [18]. Briefly, NLuc luciferase assay, Nano-Glo Luciferase Assay System (Promega, U.S.) was quantified using a Victor X3 multilabel plate reader (PerkinElmer, U.S.). MN titers were inversely proportional to the levels of NLuc activity measured.

### Western blotting for VHH characterization

The reactivity of each VHH was analyzed by Western blot under either reducing or non-reducing conditions. For this purpose, recombinant HA0 protein (1 μg protein/15 μl sample buffer per lane) was mixed either with β-mercaptoethanol (β-ME) containing sample buffer (reducing conditions), or sample buffer with no β-ME (non-reducing conditions). Samples were subjected to SDS-PAGE in a 12% gel. The proteins were blotted onto a nitrocellulose membrane (Bio-Rad, U.S.). After blocking, each VHH (4 μg/ml) was incubated separately with the membrane containing HA0, the binding was detected by the addition of an anti-6 x histidine monoclonal antibody (Qiagen, U.S.), and revealed using alkaline phosphatase-conjugated goat anti-mouse IgG. Two positive control sera were included from immune llama and immune rabbit; and one negative control of a non-relevant VHH (nr-VHH). Secondary antibodies, anti-llama peroxidase-conjugated antibody (KPL, U.S.) and anti-rabbit peroxidase-conjugated antibody (Bethyl, U.S.) were used. The reaction was developed with DAB colorimetric method.

### HA sequence and structural analysis

Total viral RNA was extracted from the supernatant of infected MDCK cells using the NucleoSpin RNA Virus kit (Macherey-Nagel, U.S.) according to manufacturer’s recommendations. cDNA corresponding to the HA and NA viral RNA (vRNA) segments, was obtained using Transcriptor First Strand cDNA Synthesis Kit (Roche, Switzerland). Influenza segments were amplified by PCR using segment-specific primers and CloneAmp HiFi PCR Premix (ClonTech, Takara, U.S.). At least 3 samples of each segment were sent for sequencing and analyzed to determine the presence of mutations. In parallel, the sequence of the highly conserved segment 7, coding for M1 and M2 proteins, was analyzed as an internal strain control. Deduced amino acid substitutions in the HA sequence were analyzed using ClustalW method of BioEdit 7.2 Molecular graphics and analyses were performed with UCSF Chimera [31]. Modeling for trimeric hemagglutinins was performed using Swiss-Model (ProMod3 3.1.1) [32].

Since the HA peptide library was based on the fragment 4 of A/California/04/2009 (H1N1) pdm09, its corresponding sequence to (GenBank accession number GQ280797) was used for epitope mapping, docking analysis, and neutralization assay.

HA protein structure homology-modelling analysis was performed in silico using the Swiss Model. The modeling of VHHs was performed using Modeller of Chimera [31] and Docking was analyzed with ClusPro [33]. The 3D models of VHH and HA were obtained using as template the published crystal structures described in the corresponding figure legend.

The docking analysis was performed by selecting the RBS on HA (PDB 3LZG) and specificity-determining loop regions CDR1-3, at <5-Å resolution. Molecular docking analysis performed using as template PDB 5XLV_C. For each VHH the docking model chosen showed the highest number of members of the cluster (reflecting the probability of the interaction taking place), as well as the lowest energy needed for the binding addressing the stability of the binding.

### Mouse experiments

The study was approved by the Institutional Animal Care and Use Committee (IACUC) of the Instituto Nacional de Tecnologia Agropecuaria, Castelar (INTA-Castelar, protocol # 26–17). The experiments were performed in biosafety level 2 rooms with negative pressure relative to adjoining facility. Specific-pathogen–free female BALB/c mice, 6–8 weeks old, were provided by the animal facility at INTA. Mice were housed in cages in rooms with controlled temperature, with food and water *ad libitum*. Mice were anesthetized by intraperitoneal (ip) injection of xylazine (10 μg/g) and ketamine (100 μg/g) before intranasal (in) VHH treatment and/or virus challenge, using a volume of 50ul, divided in 25ul/nostril. The animals were monitored for signs of distress twice daily, along the observation period. Humane endpoint was implemented immediately after finding animals showing >30% of weight loss, or behavioral signs of discomfort including decreased activity, huddling, hunched posture, and ruffled fur. Euthanasia was performed by overdose of anesthesia xylazine: ketamine given ip, followed by cervical dislocation and posterior confirmation of lack of heartbeat and/respiratory rate. No animals died before meeting these criteria. Planned euthanasia was performed at day 4 after virus challenge, lung tissue was harvested for the analysis of virus titers in lung homogenates.

The intranasal challenge with the mouse adapted virus, hu/Arg/09 ma, triggered weight loss of 30%, and signs of discomfort (S5A and S5B Fig). Regarding the *in vitro* replication, the hu/Arg/09ma variant reached similar titers to hu/Arg/09 (S5C Fig). TCID_50_ and LD_50_ of hu/Arg/09ma virus were determined using the Reed & Muench method [21]. For hu/Arg/09ma 1 LD_50_ equals 10^6.5^ TCID_50_, while for PR8 1 LD_50_ equals 10^4.5^ TCID_50_, (S6A–S6D Fig).

For all the prophylactic efficacy assays, mice (n = 5) were treated with a single administration of each specific VHH (dose 5mg/kg, unless indicated), diluted in endotoxin-free PBS with 1% (wt/vol) bovine serum albumin (BSA), non-relevant VHH (nr-VHH), mouse immune serum (dil 1:6, in PBS) (α- H1N1), or saline, 4 h before virus challenge. Body weight was monitored for 12 days after infection. The screening of VHH candidates was performed testing the prophylactic efficacy of molecules at a dose of 5 mg/kg [13, 34], against two median lethal doses (2 LD_50_) of hu/Arg/09ma. For characterization of the prophylactic efficacy, VHHs were given at a dose of 5mg/kg and the stringency of the assay was increased by applying 4 LD_50_ of either hu/Arg/09ma, or hu/PR8/34 for challenge. When hu/Arg/09 was used for the challenge, the dose given 50,000 TCID_50_, and due to the absence of weight loss, mice were monitored for 4 days (n = 5). Effective dose of VHHs was tested after treatment using either E13 with doses ranging from 100 to 1 μg per mouse (n = 5) (5 to 0.05 mg/kg); or G41 with doses of 200 μg and 100 μg per mouse (10 and 5 mg/kg) 4 h before challenge with 4 LD_50_ of hu/Arg/09ma (Table 1).

**Table 1:** Summary of the in vivo assays performed.

	Treatment		Challenge	
Assay	VHHs	Dose (mg/kg)	Virus	Challenge Dose [LD_50_(TCID_50_)]
Screening of VHHs	A5 B33 D81 D91 E13 G41	5	hu/Arg/09 ma	2 LD_50_ (10^6.81^)
Virus specific efficacy	E13 G41	5	hu/Arg/09 ma hu/Arg/09 hu/PR8/34	4 LD_50_ (10^7.12^) na (10 ^4.58^) 4 LD_50_ (10^5.11^)
Effective dose-response	E13	5 1.25 0.5 0.05	hu/Arg/09 ma	4 LD_50_ (10^7.12^)
	G41	10 5		

VHHs used for treatment and the dose prophylactic dose applied, virus used for the challenge and the corresponding infectious dose used. (na: non available).

### Viral titer in lung tissue

Lungs of infected mice were homogenized by mechanical methods using sand and a glass rod. The viral titer was determined by infection of MDCK monolayers, the presence of virus was also tested in the supernatant by hemagglutination. The viral titer was calculated by the Reed & Muench method and were expressed as tissue culture infectious doses per ml which infected 50% of the cell monolayer (TCID_50_/ml) [21].

### Statistical analysis

GraphPad Prism 5 software (version 5.01) was used for statistical analysis. Differences between groups were tested using one way ANOVA. When this test demonstrated a significant difference between groups (P< 0.05), 2 correcting methods for multiple comparisons were used (Dunnett’s and Tukey’s tests); t tests were used to compare 2 groups. Kaplan-Meier survival curves were plotted and evaluated.

Body weight analysis was performed by comparing all the treatment groups against saline treated group, at day 7 post-inoculation.

## Results

### Llama immunization and VHH library construction

**Fig 1:**
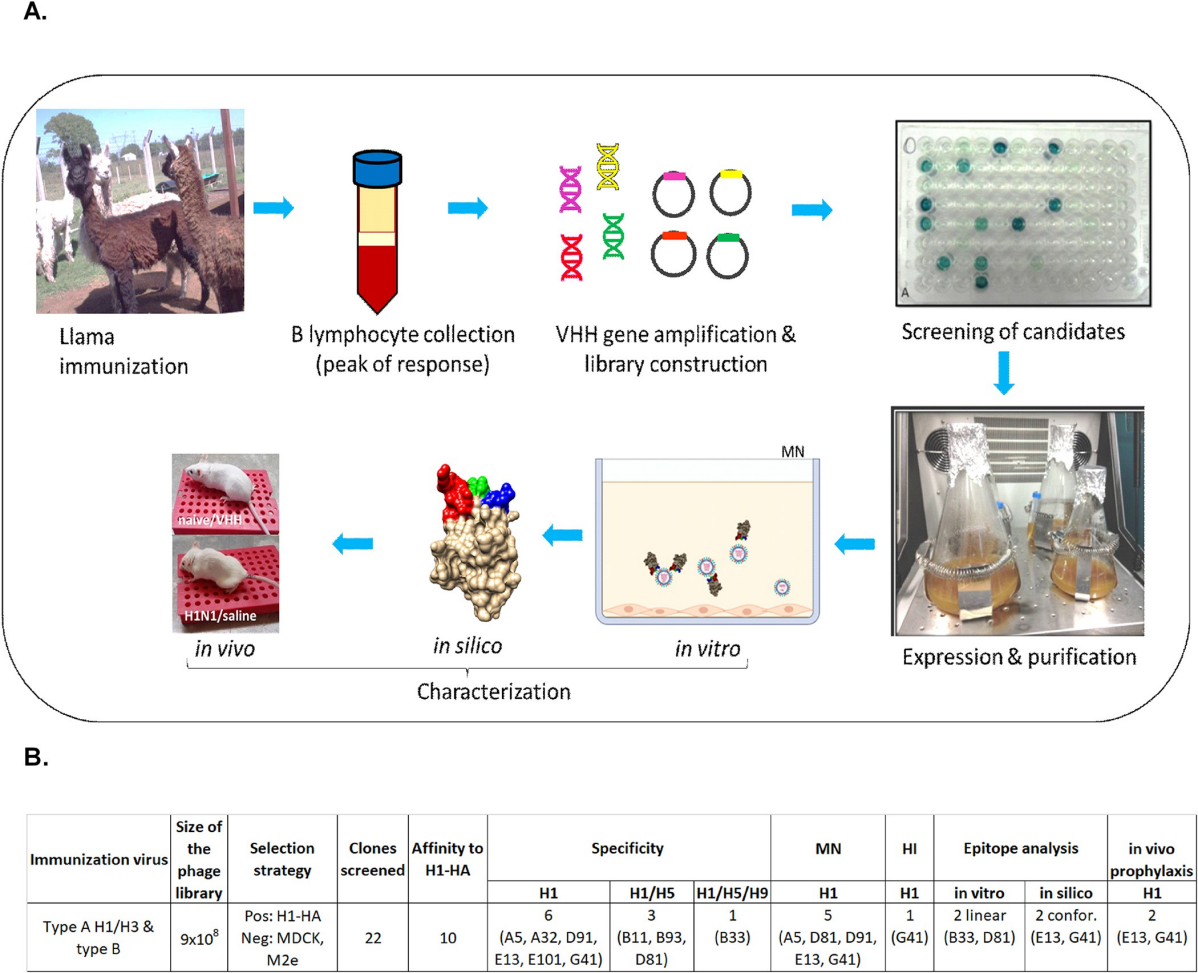
Development and characterization outline of H1N1 specific nanobodies. A. Schematic outline of the development and characterization of H1N1 specific nanobodies followed in the present report. Repeated immunization of the llama triggered the clonal expansion and selection of B cells expressing specific antibodies. From the mononuclear cells isolated from peripheral blood, the heavy chain Ig specific mRNAs were specifically amplified and cloned in phagemid plasmids, generating a VHH library. Phage display was used for the screening of the candidate VHHs, and followed by in vitro, in silico and in vivo characterizations. B. Summary of the number of clones obtained after each step of the process.

**Fig 2:**
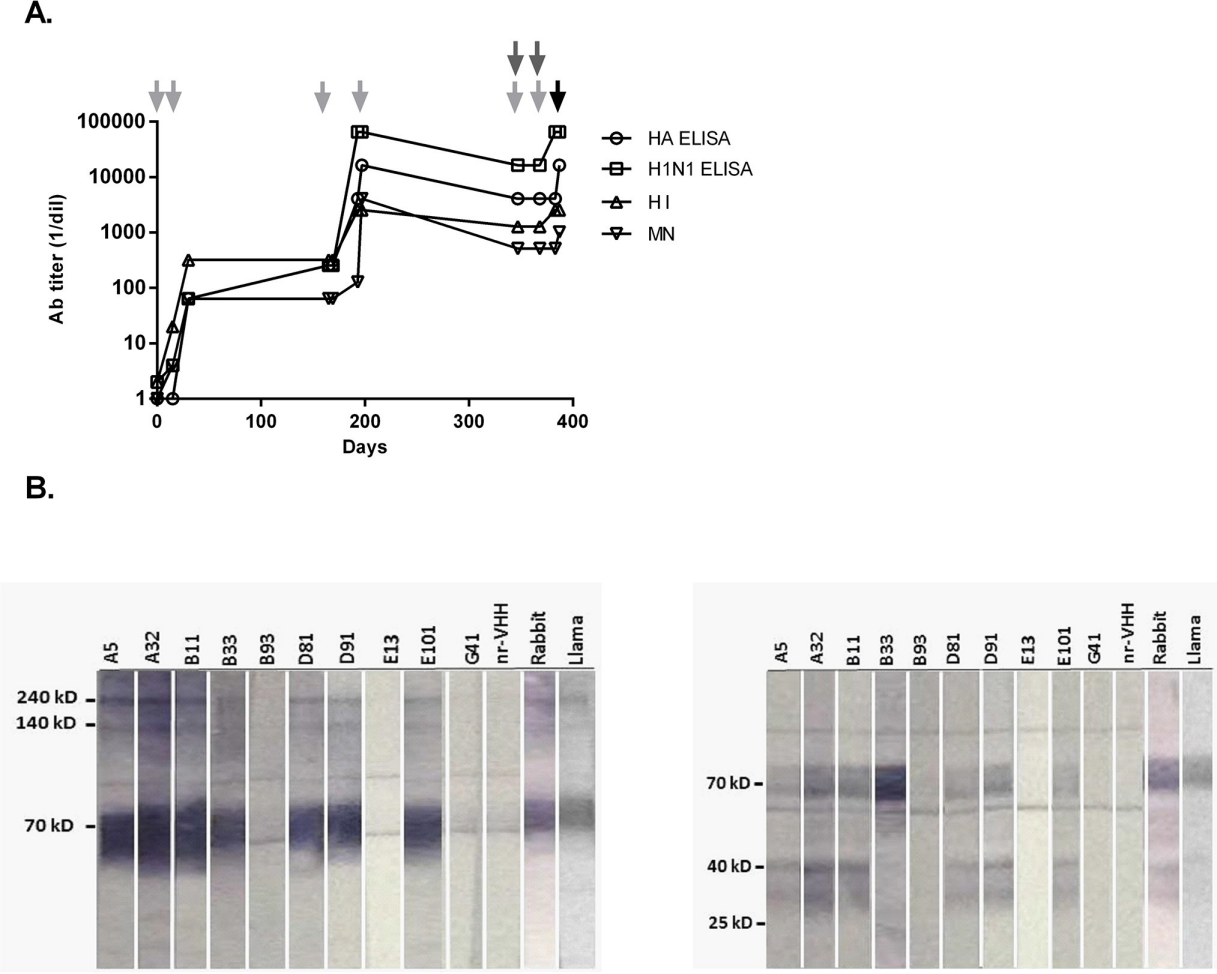
VHH development. A. Specific anti-H1N1 response in the llama serum. Six doses of monovalent vaccine containing A/California/07/2009 (H1N1) antigen are indicated with light grey arrows, and two doses of the trivalent vaccine (A/California/07/2009 (H1N1), A/Perth/16/2009 (H3N2), B/Brisbane/60/2008 are indicated with dark grey arrows. Black arrow indicates terminal bleeding. Evaluation of specific immune response to H1N1 in serum was assessed by HA ELISA; and H1N1 ELISA; hemagglutination inhibition (HI) assay; and micro neutralization (MN). Results are indicated as the inverse of the highest serum dilution with positive signal. B. VHH specific anti-HA0 reactivity. The type of epitope recognized on HA0 was evaluated by Western blot run either under non-reducing (left panel) vs. reducing conditions (with β-mercaptoethanol) (right panel). A nonrelated VHH against Norovirus was used as negative control (nr-VHH). Anti-VHH rabbit polyclonal serum was included as background control. As positive controls H1N1 rabbit and llama immune sera were included. Bands detected correspond to monomeric (70kD), dimeric (140kD) and trimeric (240kD) conformations of HA0 (70kD), HA1 (40kD), and HA2 (25kD).

Camelid derived VHHs have been developed for diagnosis and potential therapeutic applications. For influenza viruses, VHHs targeting various viral proteins, particularly the HA, have been developed. We sought to further characterize the VHH repertoire against the H1 HA subtype of influenza, in order to discover additional epitopes susceptible to therapeutic intervention against influenza. A single llama immunized with a combination of commercially available monovalent 2009 pandemic H1N1 vaccine and a trivalent influenza vaccine led to significant increase of H1N1 specific antibodies along the immunization period reaching a maximum for total antibodies after the third immunization using the monomeric vaccine, and sustained until the second immunization performed with the monomeric/trimeric vaccine regime (Figs 1A and 2A).

On day 387 post-immunization, a total of 5.3 x 10^8^ mononuclear cells were obtained for VHH library preparation (Fig 1B). After repeated immunizations using multivalent immunogens, heterosubtypic binders can be obtained and the neutralizing activity of those can be of therapeutical application. Twenty-two VHH clones were identified that recognized the recombinant hemagglutinin HA-PR8. Four clonally different candidates were chosen for further characterization based on the CDR3 sequence and yield (ranging from 0.91 to 26.92 mg/L) (Table 2, S1 Fig).

**Table 2:** VHH characterization.

VHH	Sequence	Yield	EC_50(_nM)					MN (μM)			HI (μM)
			H1N1			H5N1	H9N1	H1N1			H1N1
	CDR3	(mg/L)	hu/Arg/09	hu/Arg/09 ma	hu/PR8/34	hu/Viet/04	gf/HK/99	hu/Arg/09	hu/Arg/09 ma	hu/PR8/34	hu/Arg/09
**A5**	AATPTCGVKFQTRSYR	1.71	0.38	0.18	0.84	-	-	14.21	-	-	-
**A32**	AARRTCGPSWRSRPFD	3.03	0.34	0.32	0.75	-	-	-	-	-	-
**B11**	AARRTCGPSWRSRPFD	11.82	0.21	0.22	0.44	0.37	-	-	-	-	-
**B33**	TADAVGSTRSPDRWE	0.91	117.10	230.30	196.50	45.17	9.31	-	-	-	-
**B93**	ALDFDREYLQFWKNPRRYR	10.98	115.60	79.79	-	215.60	-	-	-	-	-
**D81**	AVTRTCGVRFATRTYT	1.98	0.56	0.20	0.89	38.65	-	2.34	0.1	0.43	-
**D91**	GATRTCGVRFVTRSYA	3.92	0.25	0.23	1.30	-	-	2.30	0.21	0.05	-
**E13**	NVQSRIVRITD	1.56	0.82	0.40	-	-	-	0.08	0.06	0.01	-
**E101**	AATPTCGVKFYTRSFR	26.92	0.34	0.24	1.09	-	-	-	-	-	-
**G41**	NGVLGTTH	14.86	3.44	2.82	0.41	-	-	0.04	0.24	0.94	0.05

Nanobody CDR3 amino acid sequences are shown. Analysis of specificity against subtypes H1, H5, and H9 from human and animal isolates. Medium effective concentration (EC_50_) was expressed as the molar concentration of VHH required to reach 50% of the specific binding. Neutralizing activity was tested against the H1 viruses used for animal work. Hemagglutination inhibition was tested against the H1 hu/Arg/09 isolate. Titers are expressed as the lowest molar concentration able to show the activity. (-) indicates lack of reaction.

Both heterosubtypic and H1-specific binders became evident after testing against subtypes H1, H3, H5, and H9. Of these, VHH B33 showed heterosubtypic binding being able to recognize H1, H5, and H9 HA subtypes with EC_50_ values of 117.10 nM, 45.17nM, and 9.31 nM, respectively. VHH D81 bound H1 and H5 HA subtype viruses, with EC_50_ values of 0.56 nM and 38.65 nM, respectively. Finally, VHHs E13 and G41 were both H1-specific binders with EC_50_ values of 0.82 nM and 3.44 nM against hu/Arg/09, respectively (Table 2), and G41 accumulated the highest number of mutations in the variable domain (S2 Fig).

### B33 and D81 exhibit heterosubtypic binding to linear and highly conserved epitopes

By SDS-PAGE western blot assay (WB) under non-reducing conditions, 7 out of 10 nanobodies (A5, A32, B11, B33, D81, D91, E101) were found to recognize linear epitopes on the three conformations of HA0, trimeric (approximately 240 kD), dimeric (approximately 140 kD) and monomeric (approximately 70 kD). After separating HA1 from HA2 under reducing conditions WB (Fig 2B), the data suggested that 6 of the VHHs recognize epitopes located on HA1 (40 kD), while B33 recognizes an epitope on HA2 (25 kD) (Fig 2B and S3 Fig).

**Fig 3:**
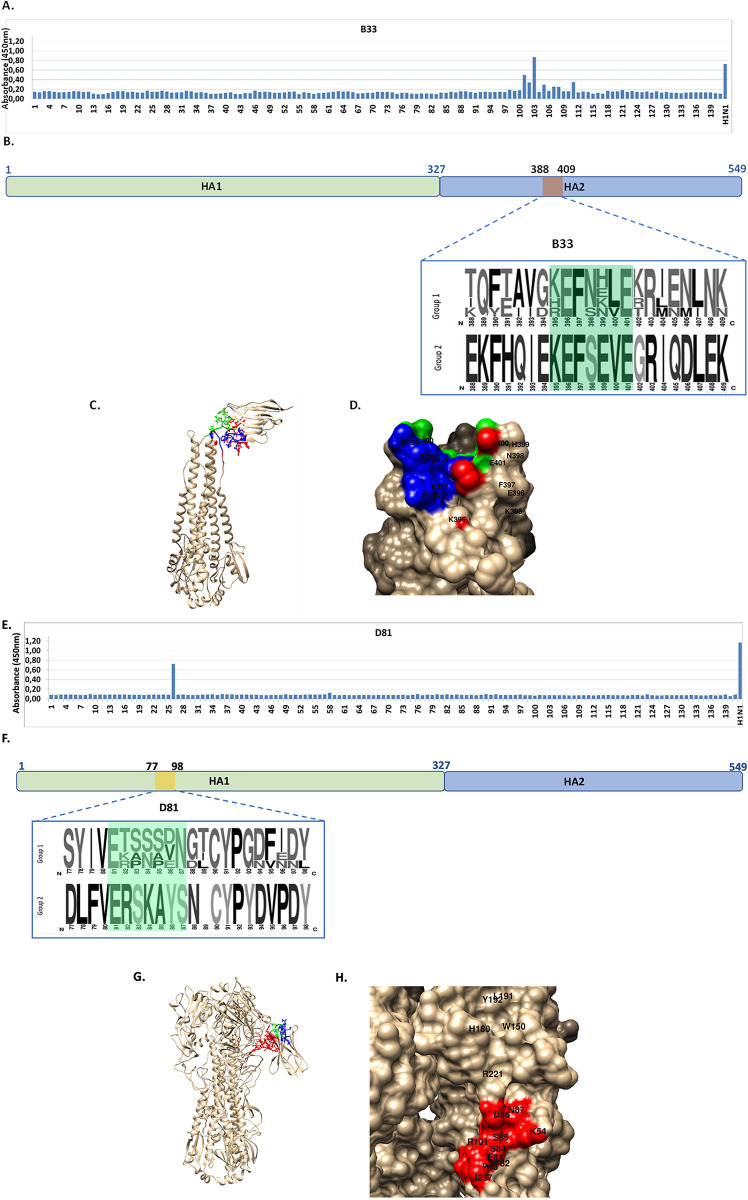
Mapping linear epitopes recognized by B33 and D81. A and E, ELISA with overlapping peptides corresponding to hemagglutinin HA of A/California/04/2009 (H1N1) pdm09. A concentration of 4 ng/well of peptide individually detected by 20 ng/well of VHH. Results are expressed as the absorbance measured at 450 nm. B and F, Epitope sequence showing the variability of the HA sequences analyzed by LOGOS for viruses belonging to group 1 ((H1N1) hu/Ca/09ma; hu/Arg/09; hu/Arg/09ma; hu/PR8/34; (H5N1) hu/Viet/04, and (H9N1) gf/HK/99, and group 2 (H3N2) hu/Perth/09 used in the ELISA. C and G, Ribbon representation, D and H. Surface representation with detail of the residues involved in the specific binding. Molecular modelling of the VHH and docking analysis against hu/Ca/09 HA (PDB structure 3LZG, at <5-Å resolution). The crystal structures used as templates for 3D modelling of B33 were 5F1K_C, 6GS1_H, 4WEU, and for D81 were 5VXK_B, 6UL4_B, 5JAB_B, 6GWN_C. Relevant amino acid residues for interaction are color coded: CDR1 green, CDR2 blue, and CDR3 red.

We analyzed the capability of B33 and D81 to recognize linear epitopes on an array of overlapping HA peptides. When B33 was tested, a positive reactivity was obtained for peptides located on HA2 covering positions 384–398 (EKMNTQFTAVGKEFN), 388–402 (TQFTAVGKEFNHLEK), and 392–406 (AVGKEFNHLEKRIEN), but no reactivity was observed for the flanking overlapping peptides. These data suggested residues KEFNHLEKRIEN as critical for binding the epitope with B33 under the conditions of the assay (Fig 3A). After analyzing the docking of B33 to the hemagglutinin, the model with the highest probability (123 members) and the lowest energy (-175.9), brought us to the conclusion that residues KEFNHLE (position 395–401) would be critical for binding on HA2, with this interaction being shared by the three CDR domains of the VHH (Fig 3B–3D). On the other hand, for D81, the putative epitope was found located on HA1 between positions 84–98 (SSDNGTCYPGDFIDY) while no reactivity was observed for the flanking overlapping peptides (Fig 3E). After analyzing the docking of D81 to the hemagglutinin, the model with the highest probability (136 members) and the lowest energy (-124.9), brought us to the conclusion that residues ETPSSDN (positions 81–87) would be critical for binding on HA1. Interestingly, this analysis revealed that the binding would include residues K54, R92, R221, and I267; with all these interactions being established exclusively by the CDR3 domain of the VHH (Fig 3F–3H).

#### E13 and G41 exhibit strong neutralizing efficacy in vitro and in vivo

Five VHHs were found with neutralizing activity (A5, D81, D91, E13 and G41) against hu/Arg/09, hu/Arg/09ma and hu/PR8/34 (Table 2). E13 and G41 showed the highest neutralizing activity against hu/Arg/09 among the candidates. Furthermore, E13 showed a neutralizing activity towards all the H1N1 isolates from human and animal origin evaluated, with a titer ranging between 0.01 μM and 0.08 μM (Table 3). Additionally, only G41 showed capability of inhibiting viral hemagglutination towards hu/Arg/09 (HI titer of 0.05 μM) (Table 2), suggesting that the binding of the G41 completely blocks.

**Table 3:** E13 and G41 H1-specific neutralization activity.

VHH	Neutralizing activity (μM)				
	H1N1				H3N2
	hu/Bri/07	hu/Ca/09 ma	rt/Del/09	sw/SD/18	ty/OH/04
**E13**	0.04	0.04	0.01	0.04	-
**G41**	0.04	0.02	0.04	-	-

E13 and G41 were analyzed in their capability to further neutralize viruses H1N1 hu/Bri/07, hu/Ca/09 ma, rt/Del/09, and sw/SD/18. A subtype control H3N2 represented by ty/OH/04 was added to the analysis. Values are expressed as the lowest VHH molar concentration showing positive reaction. (-) indicates lack of reaction.

The H1 specific neutralizing efficacy for E13 and G41 was reinforced by an independent assay using NLuc activity as a readout of the neutralizing activity (S4A Fig). Phylogenetic analysis showed sequence disparities between viruses. While pandemic viruses maintain close similarities among them (clade 1A3.3.2), they differentiate from the recent sw/SD/18 (clade 1A.2), as well as from the pre-pandemic viruses hu/PR8/34 and hu/Bri/07 (clade 1B.2), while the avian viruses rt/Del/09 has a common ancestor which is different from all the above (S4B Fig). All together, these data indicate that the tri-dimensional conformation of the epitope recognized by E13 would be maintained along all these clades.

Undoubtedly, for E13 and G41, the failure of detection in assays in which the protein was subject of denaturation confirmed the conformational nature of the epitopes recognized. Thus, the *in-silico* analysis of the VHH footprints was performed, allowing for the theoretical modeling of the contacts of E13 and G41 to HA1. Interestingly, the models obtained would suggest that each VHH independently recognizes the conformational domain located between residues 130 and 238, although establishing contact with a particular set of amino acid residues.

**Fig 4:**
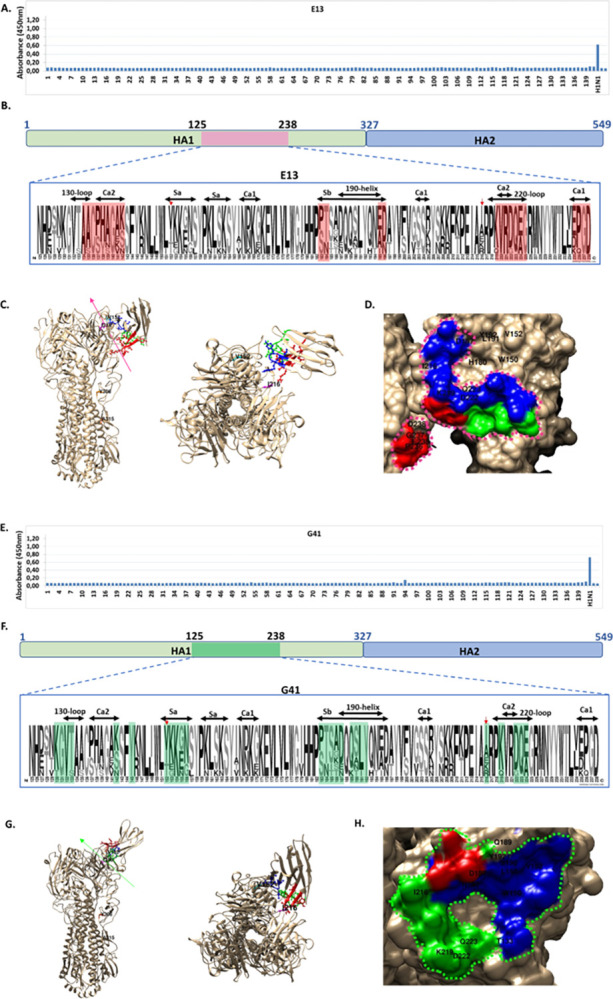
Deducing the structure of the conformational epitopes recognized by E13 and G41. A, E, Peptide array analysis. B, F, VHH deduced epitope footprint on an HA monomer. Detail of the amino acid diversity in the domain of residues involved in the VHH-HA binding, analyzed by LOGOS. The H1N1 viruses used in neutralizing assays were: hu/Ca/09; hu/Ca/09ma; hu/Arg/09; hu/Arg/09ma; hu/PR8/34; hu/Bri/07; rt/Del/09; and sw/SD/18. Residues involved in the interaction with E13 and G41 are indicated in pink or green boxes, correspondingly. Relevant antigenic domains and RBS structures are labeled. C, G, Ribbon representation for HA interacting with the VHH. Side and top views. Residues on positions 152, 216, 306, 315 are labeled. D, H, Surface representation with detail of the residues involved in the specific binding. The shape of the putative domain of interaction is indicated by colored broken lines. Amino acid residues critical for the interaction are indicated in color code: CDR1 is colored in green, CDR2 in blue, and CDR3 in red. For the 3D modelling of E13 crystal structures of 6H7L_C, 5HVG_B, 6H7J_C were used as templates, and for G41 structures of 5XLV_C,5JQH_C, 4H3J_A. The model with the highest probability for E13 showed 106 members, while in the case of G41 the cluster showed 232 members.

E13 would bind a total of 24 residues, 20 of those distributed along a gap of 107 residues in proximal positions to the RBS on one monomer of HA0, and 4 residues of a neighbor monomer of HA0. The residues critical for the interaction would be AACPHAGAK (134–142) on Ca2, ST (183,184) on Sb, AD (195 and 196) on 190 Helix, KVRDQEG (219–225) on 220Loop, and residues EPGD (235–238) on Ca1 of the neighbor HA0 monomer (Fig 4A–4D). The modeling *in silico* of these interactions showed the lack of participation of HA1 residues on positions 152 and 216 earlier described as susceptible for amino acid substitution. Interestingly, the epitope for E13 would map a C-shaped domain of residues, partially interfering the access to the RBS (Fig 4D) in one HA0 monomer and would extend to four residues (EPGD) on a neighbor monomer, which could potentially stabilize the E13-HA complex.

G41 would recognize a total of 25 residues distributed along 94 amino acid residues of the RBS on the same monomer of HA0. The positions critical for the interaction would be positions KGVT (130–133) on Loop130, residues K and Y (142 and 145) on Ca2, VKKGN (152–156) on Sa, STSAD (183–187) on Sb, QSLY (189–192) on 190 Helix, residue I (216) and K (219), and DQE (222–224) on Loop 220. These interactions showed the direct participation of residues on positions 152 and 216, which after mouse lung adaption were susceptible to amino acid substitution described above (Fig 4E–4H). Interestingly, the epitope for G41 would map in an O shaped domain of residues establishing direct contact with residues of the RBS structural domains, suggesting that the binding of the VHH would occlude the access to the RBS (Fig 4H).

**Fig 5:**
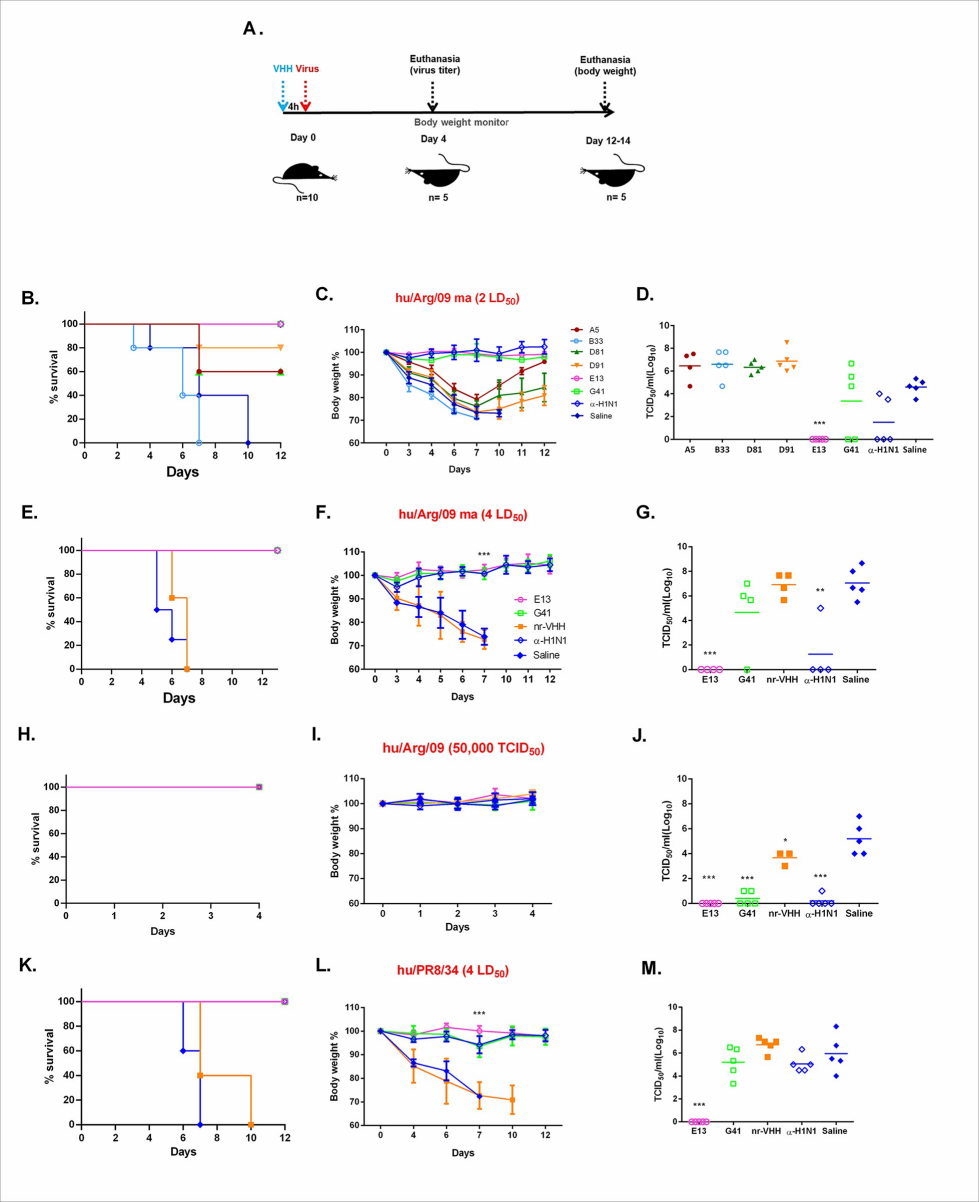
Prophylactic efficacy against H1N1 virus challenge. A. Experimental design. B, C, D, Screening of VHH candidates using hu/Arg/09 ma (2LD_50_). E, F, G challenge with hu/Arg/ 09 ma (4LD_50_); H, I, J, challenge with hu/Arg/09 (viral dose 50,000 TCID_50_); K, L, M challenge with hu/PR8/34 ma (4LD_50_). B, E, H, K, Survival curve. C, F, I, L, Body weight represented as the mean percentage of the initial body weight in each group. D, G, J, M, viral titer determined by TCID_50_ on MDCK cell monolayers. Female mice BALB/c were treated by intranasal route with 5 mg/kg of specific VHH, non-related VHH (nr-VHH), hu/Arg/09 mouse immune serum (dil 1/6) (α- H1N1), or saline, 4 h before viral challenge with virus according to the label. Viral titer in lung homogenates was analyzed on day 4 after inoculation (n = 5). Body weight was monitored for 12 days after infection. Error bars represent standard deviations. Symbols are the same for all panels. One-way ANOVA; Dunnett’s Multiple Comparison Test vs Saline group; *: p<0,05; **: p<0,01; ***: p<0,001.

After the screening of VHHs for their prophylactic efficacy, against hu/Arg/09ma, E13 and G41 became the candidate molecules for further *in vivo* experiments. E13 treated animals showed full protection against viral infection, with viral loads below the limit of detection in the lungs, no clinical signs nor body weight loss. On the other hand, G41 treated animals showed partial protection since 60% of the mice exhibited viral titers in lung tissue ranging between 10^4.66^ and 10^6.66^, with no body weight loss (Fig 5C and 5D). Mice treated with either A5, D81, or D91 shown weak protection with weigh recovery by the end of the assay, while the treatment with B33 gave no prophylactic effect under the conditions of the assay.

As shown in Fig 5B–5D the VHHs B33 and D81 evidenced none or weak potential for their therapeutical application against the H1N1 virus hu/Arg/09, in their monomeric form. While, the treatment with D81 improved survival as animals were able to recover from weight loss, B33 treatment did not show any improvement measured by the three major indicators of prophylactic effect: either by survival, weight loss nor virus titer in the lung.

In view of the prophylactic efficacy found for G41 and E13 using 2 LD_50_ of hu/Arg/09 ma, we evaluated the efficacy in a more stringent scenario using 4 LD_50_. Additionally, we evaluated the protection with two other viruses, hu/Arg/09 and hu/PR8/34. The results showed total protection for mice treated with E13 (5 mg/kg) with undetectable infectious virus for all the viruses assayed, and no body weight loss (Fig 5F). The animals treated with G41, no significant reduction in viral titers detected with no body weight loss (Fig 5G) indicating partial protection. More importantly, when G41 treated mice were challenged with the wildtype hu/Arg/09 virus (Fig 5I), showed significant protection since 100% of the mice with viral titers like those observed for α-H1N1 serum treated animals (Fig 5J). Furthermore, mice treated with G41 and challenged with hu/PR8/34 showed no significant reduction in viral titers, and no body weight loss (Fig 5M). These results reinforced the hypothesis that the prophylactic effect of G41 is partially lost after virus adaption to mouse.

**Fig 6:**
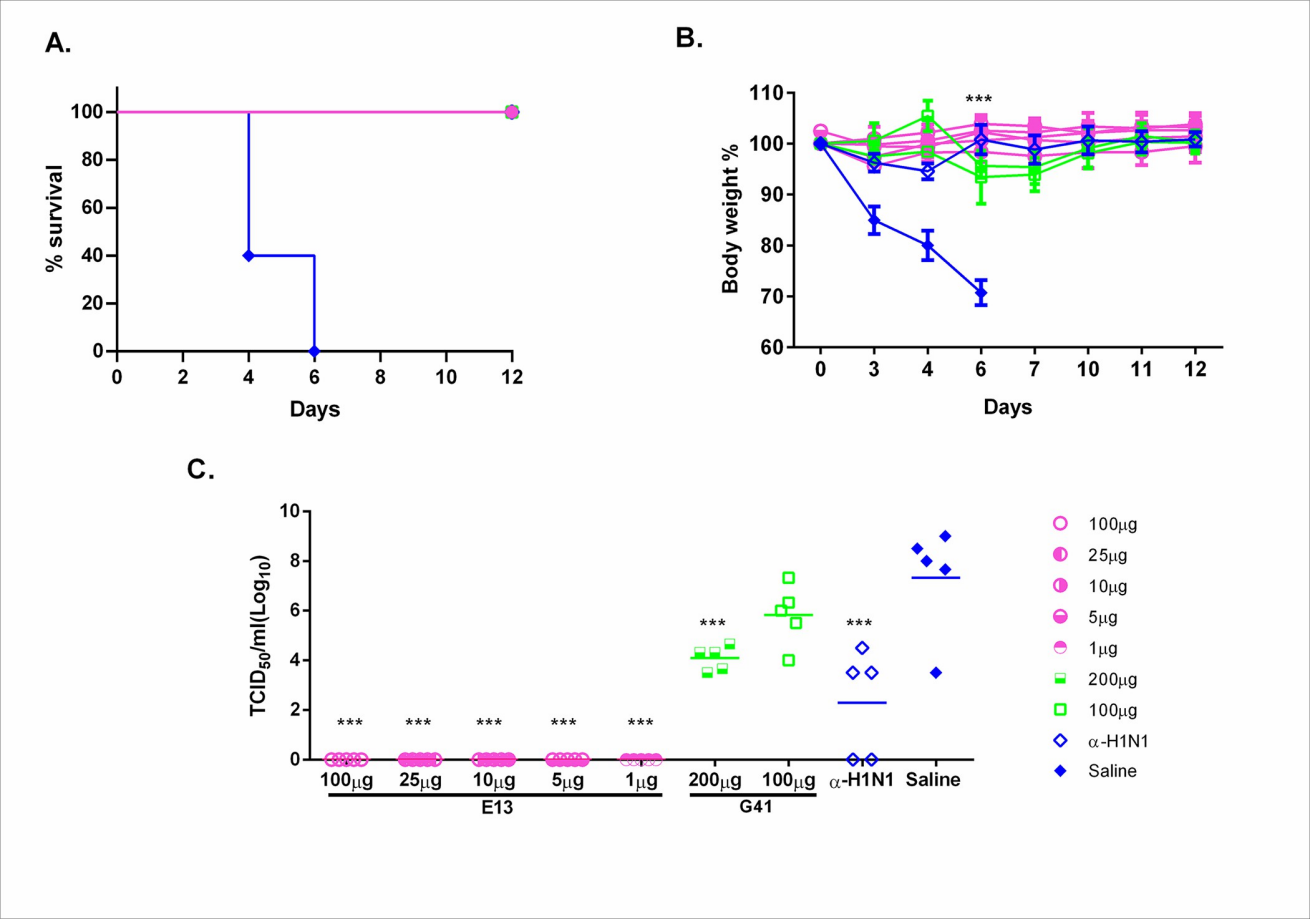
Effective dose of E13 and G41. A, Kaplan-Meier survival curve for the analysis of effective dose. B, Body weight after infection is represented as the mean percentage of initial body weight for all mice in each group. C, Viral titer in lung tissue was analyzed on day 4 post viral challenge. Error bars represent standard deviations of the mean. BALB/c mice (n = 5) were given saline, or doses of 100, 25, 10, 5, 1 μg of E13, and 200 or 100 μg of G41, or mouse immune serum (α-H1N1); 4 h later mice were challenged with hu/PR8/34 virus (4LD_50_). Body weight was monitored for 12 days. Symbols are the same for panels a, b, and c. One-way ANOVA; Dunnett’s Multiple Comparison Test vs saline group; *: p<0,05; **: p<0,01; ***: p<0,001.

To look more in detail the effective concentrations needed for E13 and G41, we established minimum effective dose for both neutralizing nanobodies. Treatment with E13 showed full protection even when 0.05mg/kg was administered intranasally with undetectable virus titer in lung homogenates and preventing weight loss. Treatment with G41 showed only partial protection at both doses 5mg/kg and 10mg/kg with average viral titers of 10^5,8^ and 10^4^, respectively, while abrogated weight loss in both treatment groups (Fig 6).

## Discussion

In order prevent the next influenza pandemic, it becomes critically important to generate type A influenza therapeutic countermeasures. Recent antigenically novel H1-expressing viruses have been the product of transmission from animals into humans, and two of the last four influenza virus pandemics were caused by H1N1 viruses [35]. Despite the lack of evidence for sustainable transmission of H5 and H9 viruses among humans, sporadic human infections have been reported in multiple countries, addressing them as a significant pandemic threat [36, 37]. Despite an overall efficacy, no higher than 60%, vaccination remains essential to prevent or reduce the severity of the disease [38]. In this context, recombinant monoclonal nanobodies could be of value either for prophylactic treatment of vulnerable individuals during influenza seasons, for the treatment of severe cases, or mitigation of pandemic events.

Herein we described the development of recombinant monoclonal nanobodies using repeated immunizations as an approach of choice in order to maximize B cell clone diversity through somatic hypermutation in germinal centers leading to affinity maturation of the immunoglobulins [39]. As expected, a high neutralizing titer was observed in the llama serum although, the neutralizing VHH were not heterosubtypic binders as other authors have described [13, 14, 40, 41]. The reason behind these binding properties could be associated to the repeated administration of H1N1 antigen, since repeated immunizations with a single antigen has been shown to decrease cross-reactivity [39, 42]. From our results, G41 could be interpreted as the result of high accumulation of mutations detected in the V-domain mostly in the FR domain, which in turn refined the specificity towards a hu/Arg/09, along the multiple cycles of maturation. In the same line of thought, a lower number of maturation cycles may have determined the pan-H1 binding for E13.

Among the twenty-two heavy chain H1N1 specific antibodies were selected, B33 was characterized as an heterosubtypic binder able to detect subtypes belonging to phylogenetic group 1 H1, and H5, H9. Heterosubtypic binding for antibodies recognizing conserved linear epitopes on HA2, as B33 have been described by other authors [43], although the epitope bound by B33 remains hidden under the globular HA1. Most probably, the ability of B33 to access the specific domain is favored by the features of flexibility and solubility described for VHHs. Meanwhile, heterosubtypic binding for antibodies recognizing conserved linear epitopes on HA1, as D81, are much rarely found. D81 binds a total of 11 residues, 7 which are continuous among themselves and another 4 residues scattered along 213 positions. Apparently, the ETPSSDN domain dominates the interaction, thus D81 behaves as if the specific epitope was linear. In order to verify the hypothesis, site directed mutagenesis studies would be necessary. From our results, B33 and D81 remain as two heterosubtypic binders which combined with other neutralizing VHHs, could broaden and strengthen the reactivity of the chimera, as it has been described by other authors [14, 44].

Although, several broad reactive nanobodies have been described for influenza viruses, the information about their capability of recognizing viruses originated from the most relevant animal species in the influenza epidemiology remained unclear up to now. These features become relevant due to the increasing consensus on the risk represented by animal species as potential source of emerging influenza viruses.

Among the VHHs with neutralizing activity VHHs E13 and G41 showed high efficacy ranging between 0.94 and 0.01μM when tested against seven H1N1, including isolates human, avian, and swine species, spanning more than 80 years of viral drift. From those results, the conservation of the conformational backbone became evident, despite the high variability of the hemagglutinin sequence detected among the viruses analyzed.

From the *in-silico* analysis, most of the amino acid residues bound by G41 are located either proximal or on the RBS. This type of binding can occlude the interaction with the sialic acid, aiding both neutralization of virus infection and inhibition of hemagglutination. Thus, G41 treatment protected from virus replication in cell culture and mice treated with 5mg/kg four hours prior to hu/Arg/09 virus challenge. However, limited efficacy towards hu/Arg/09ma and hu/PR8/34 was observed for G41, showing partial protection as described for other VHHs in the literature [13]. The partial prophylactic effect observed for G41 against the virus after mouse adaption of hu/Arg/09 made evident the critical role imposed by the substitution residues at positions 152 and 216. Additionally, docking analysis suggested the direct interaction of G41 with residues V152 and I216 among the 24 residues bound by G41, in the proximity of the RBS. We hypothesize that the change of these two residues could have affected the stability of the binding between the VHH and HA. Further studies would be needed to confirm these hypotheses and dissect the molecular determinant that favor G41 binding.

Interestingly, the differences on hemagglutination inhibition between VHHs are given by the differences on the epitope footprint. On one hand, G41 would bind residues located on the surrounding area of the RBS, thus interfering the interaction with the sialic acid on the target cell. On the other hand, E13 would bind residues proximal to the RBS on one side, interacting with two HA0 monomers simultaneously, which would maintain the capability of binding to the sialic acid on the target cell, potentially interfering with further stages of the viral cycle. This type of interaction is reflected by the absence of HI activity for E13 observed in our studies. Of note, the interaction with a second HA monomer that could potentially stabilize the binding to the HA molecule on the virus envelope, and thus, could support the sterilizing capacity observed *in vivo*. Further mutational and binding kinetics studies on E13 could confirm these hypotheses.

Although E13 efficiently neutralized all H1N1 viruses tested, the lack of detection of E13, to hu/PR8/34 may be addressing a binding mechanism which could be differential compared to other VHHs since, according to our analysis, it would take the interaction of two HA0 monomers.

A 100-fold enhanced neutralizing efficacy has been previously described for antibodies simultaneous binding of multiple independent epitopes, for multimeric nanobodies [13, 45]. Despite the monomeric structure of E13, the observed prophylactic efficacy could be explained by the simultaneous binding to two independent HA0 molecules of the HA trimer described by the theoretical docking analysis. Thus, molecular crystal structure analysis would clarify the results obtained from the theoretical representation of the contacts for the VHHs with the target molecule.

Among the neutralizing VHH candidates, E13 showed the highest efficacy on neutralizing H1 subtype viruses from human and animal origin, including the newly isolated A/swine/South Dakota/2018 (H1N1). Although, E13 showed similar *in vitro* neutralizing activity to the described by other authors for H1N1 HA specific VHHs, the level of efficacy observed *in vivo* was surprising [14, 43]. E13 conferred sterilizing protection even when administered in the lowest dose tested (0.05 mg/Kg) to mice challenged with H1N1 viruses independently of the challenge dose used (2 or 4 LD_50_). Earlier reports showed absence of infectious virus in the lung of mice after the treatment with specific antibodies and posterior virus challenge. A dimeric VHH, containing a duplicated binding site, against H5N1 hu/Viet/04 HA, did prevent animals from infection if administered at a dose of 1.5 mg/kg, while the monomeric form of the VHH did not [13]. Side by side comparison studies would be needed in order to test the relative protection degree exerted by E13 and the previously described molecules. To our knowledge, none monomeric VHH has been described before able reduce H1 subtype influenza virus replication to undetectable levels in the lung of mice, at a dose of 0.05mg/kg.

Long spanning epitope footprints have been described previously for antibodies obtained from individuals with multiple expositions to the virus, either by infection and/or vaccination [46, 47]. Interestingly, the hypothesized epitope footprints for both G41 and E13 would partially coincide with the Sa antigenic site described to be bound by the human 2D1 derived from a 1918 Spanish flu survivor (spanning positions 125–129 and 157–169). G41 would directly bind residues 162–166 among the 25 positions of the deduced epitope, while E13 would not directly bind the residues of the Sa although the domain is included in the region spanned by the conformational interaction [46]. Furthermore, both G41 and E13 would bind the Sb antigenic site described as part of the epitope bound by the human CH65 derived from a subject immunized with a trivalent influenza vaccine [47]. These data would suggest that the long and repeated immunization of the llama, together with the combination of monovalent and trivalent vaccines allowed for the selection of broad H1 neutralizers.

The potential emergence of escape variants in the clinical setting, could represent a serious risk that needs to be considered for any new antiviral immunotherapeutic agent. The combination of antibodies directed against different epitopes is the strategy frequently taken to avoid the development of escape mutants when the treatment includes the administration of repeated doses. Although, the analysis of potential emergence of escape mutants remains to be addressed, the pan-H1 neutralizing activity observed, together with the highly conserved conformational epitope and the deduced epitope footprint, would strongly support the testing of E13 as a therapeutical agent for global distribution.

We have earlier described a side-by-side comparison of prophylactic efficacies between *E*. *coli* and *P*. *pastoris* recombinant E13 and improvements on the yield of the purification process are in progress [34].

In this study we have selected and characterized E13, a pan-H1 VHH with a potent prophylactic activity in vivo, which could be further developed as a therapeutical tool for global implementation. The therapeutical efficacy of E13 against IAV H1N1, in a preclinical setting and its potential translation to the clinics should be further explored.

## Supporting information

S1 Table Summary of viruses used for VHH characterization. (PDF)

S1 Fig VHH protein analysis. (PDF)

S2 Fig VHH sequence analysis. (PDF)

S3 Fig VHH specific reactivity to HA0. (PDF)

S4 Fig E13 and G41 neutralizing activity against viruses of subtypes H1 and H3. (PDF)

S5 Fig Pathogenicity of hu/Arg/09 along mouse adaption process. (PDF)

S6 Fig Median lethal dose (LD_50_) of viral strains used for challenge. (PDF)

S1 Dataset Minimal data sets (https://osf.io/kw5r9/). (TXT)

S1 Raw images (PDF)
