# Supplementary material for: Anti-hemagglutinin monomeric nanobody provides prophylactic immunity against H1 subtype influenza A viruses
Source: PLoS One. 2024 Jul 10;19(7):e0301664. doi: 10.1371/journal.pone.0301664 (PMC11236207; doi:10.1371/journal.pone.0301664)

Species of origin	Phylogenetic group	Subtype	Origin	Virus name	Abbreviation	Isolation reference	Analysis
human	1	H1N1	isolate	A/Argentina/017/2009pdm (H1N1)	hu/Arg/09	2009 (Pdm)	ELISA/MN/Prophyl
human	1	H1N1	lab passage	A/Argentina/017/2009pdm (H1N1) ma	hu/Arg/09 ma	2009 (Pdm)	ELISA/MN/Prophyl
human	1	H1N1	lab passage	A/PuertoRico/8/1934 (H1N1)	hu/PR8/34	1934 (pre-Pdm)	ELISA/MN/Prophyl
human	1	H1N1	isolate	A/Brisbane/59/07(H1N1)	hu/Bri/07	2007 (pre-Pdm)	MN
human	1	H1N1	lab passage	A/California/04/09 (H1N1) ma	hu/Ca/09 ma	2009 (Pdm)	MN
avian	1	H1N1	isolate	A/Ruddy Turnstone/Delaware/300/09 (H1N1)	rt/Del/09	2009 (Pdm)	MN
swine	1	H1N1	isolate	A/swine/South Dakota/2018 (H1N1)	sw/SD/18	2018 (post-Pdm)	MN
human	1	H5N1	rev genetics	A/Vietnam/1194/2004 (H5N1)	hu/Viet/04	2004 (pre-Pdm)	ELISA
avian	1	H9N1	rev genetics	A/guinea fowl/Hong Kong/WF10/1999 (H9N1)	gf/HK/99	1999 (pre-Pdm)	ELISA
human	2	H3N2	isolate	A/Perth/16/2009 (H3N2)	hu/Perth/09	2009 (Pdm)	ELISA/MN
swine	2	H3N2	isolate	A/swine/Argentina/CIP051-A2/2008 (H3N2)	sw/Arg/08	2008 (pre-Pdm)	MN
avian	2	H3N2	rev genetics	A/turkey/Ohio/313053/2004 (H3N2)	ty/Oh/04	2004 (pre-Pdm)	MN
equine	2	H3N8	isolate	A/eq/Palermo/E-2345-1/12) (H3N8)	eq/Arg/12	2012 (post-Pdm)	MN

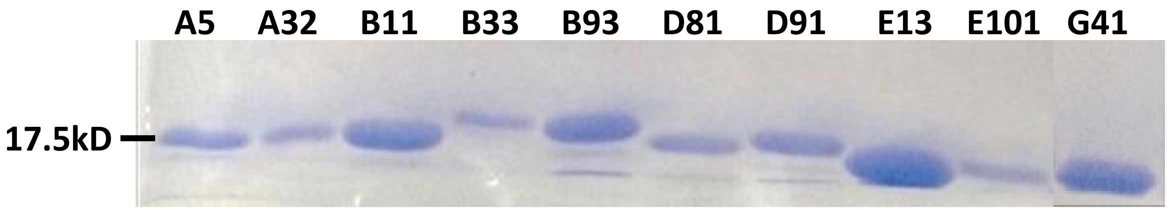
A5
A32
B11
B33
B93
D81
D91
E13
E101
G41
17.5kD

A.

	*Allele (Identity %)		# aa changed		Total mutations in V-DOMAIN
VHH	VH-GENE	JH-GENE	CDR1-CDR2	FRs	
A5	IGHV3S2*01 (79.4%)	IGHJ4*01 (92.9%)	6	14	20
A32	IGHV3S1*01 (81.0%)	IGHJ5*01 (84.6%)	9	10	19
B11	IGHV3S1*01 (80.0%)	IGHJ5*01 (84.6%)	9	10	19
B33	IGHV3S1*01 (76.5%)	IGHJ2*01 (100%)	11	11	22
B93	IGHV3S2*01 (75.0%)	IGHJ4*01 (92.9%)	6	18	24
D81	IGHV3S2*01 (76.3%)	IGHJ4*01 (92.9%)	11	12	23
D91	IGHV3S1*01 (81.2%)	IGHJ4*01 (85.7%)	7	8	15
E13	IGHV3S3*01 (80.2%)	IGHJ2*01 (100%)	7	10	17
E101	IGHV3S2*01 (80.4%)	IGHJ3*01 (90.9%)	6	13	19
G41	IGHV3S3*01 (69.5%)	IGHJ4*01 (91.7%)	8	19	27

B.

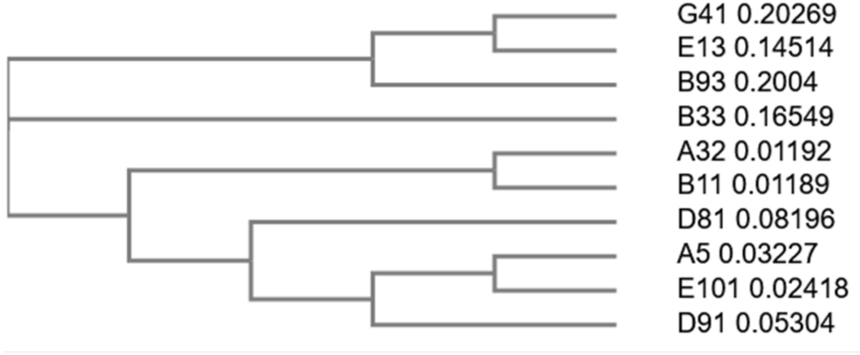

Barbieri et al. S3 Fig

A. B.

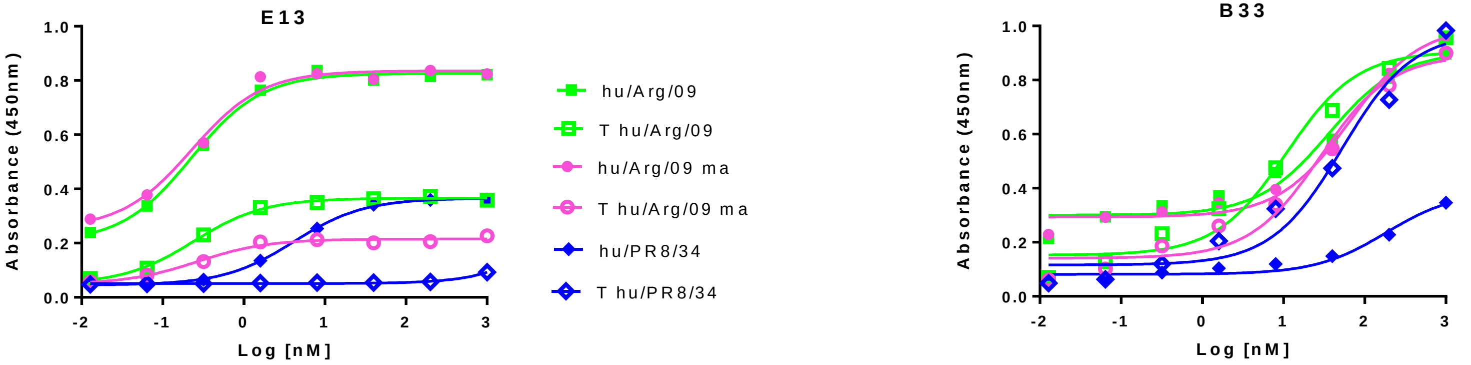

Barbieri et al. S4 Fig

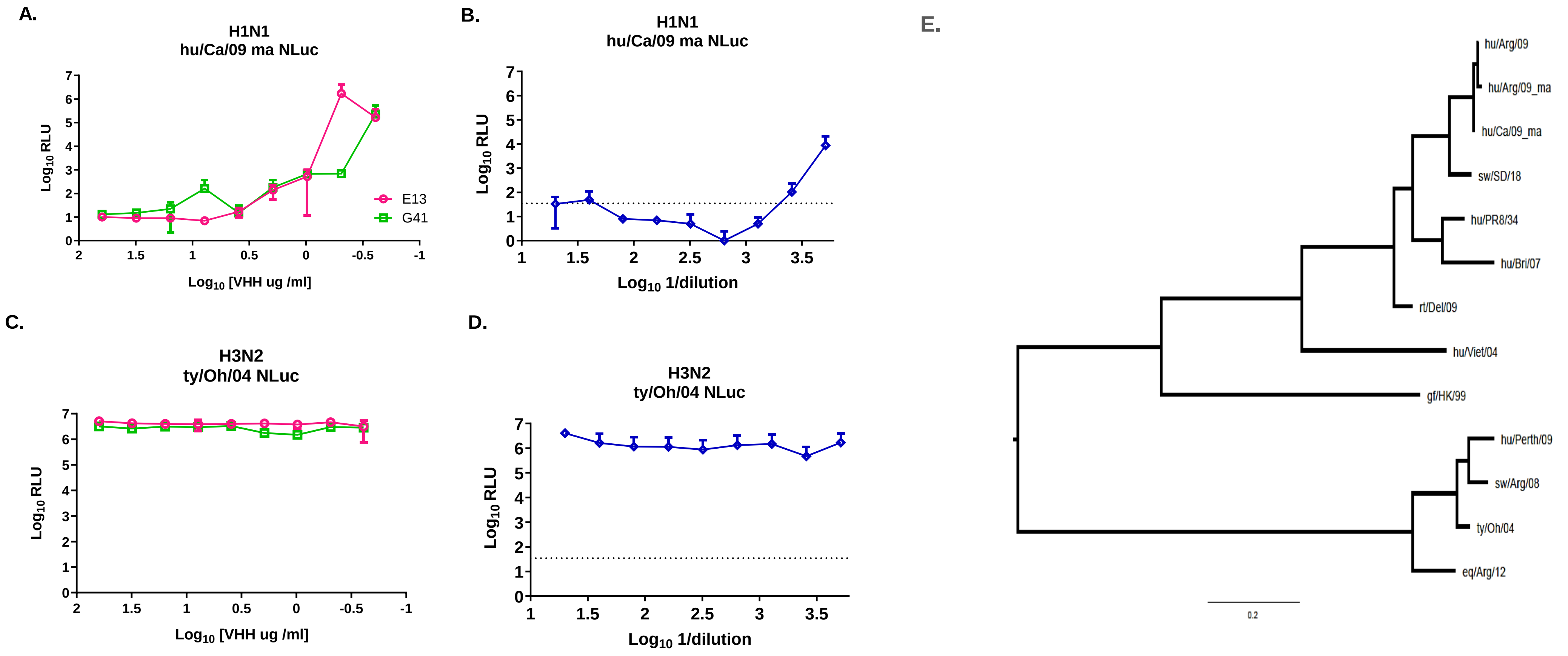

Barbieri et al. S5 Fig

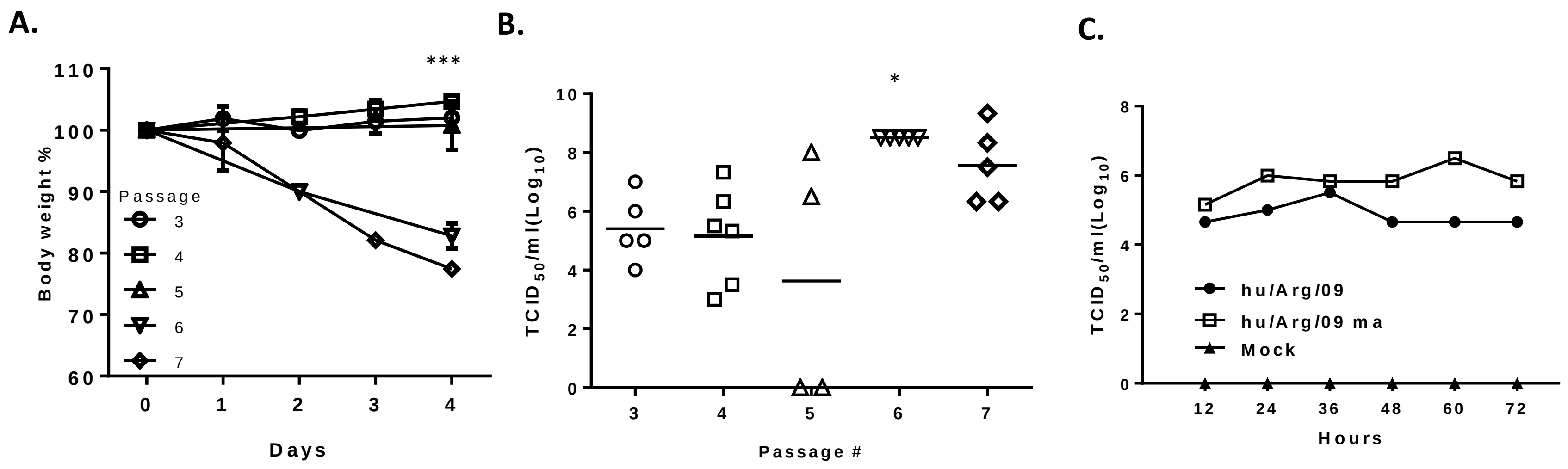

Barbieri et al. S6Fig

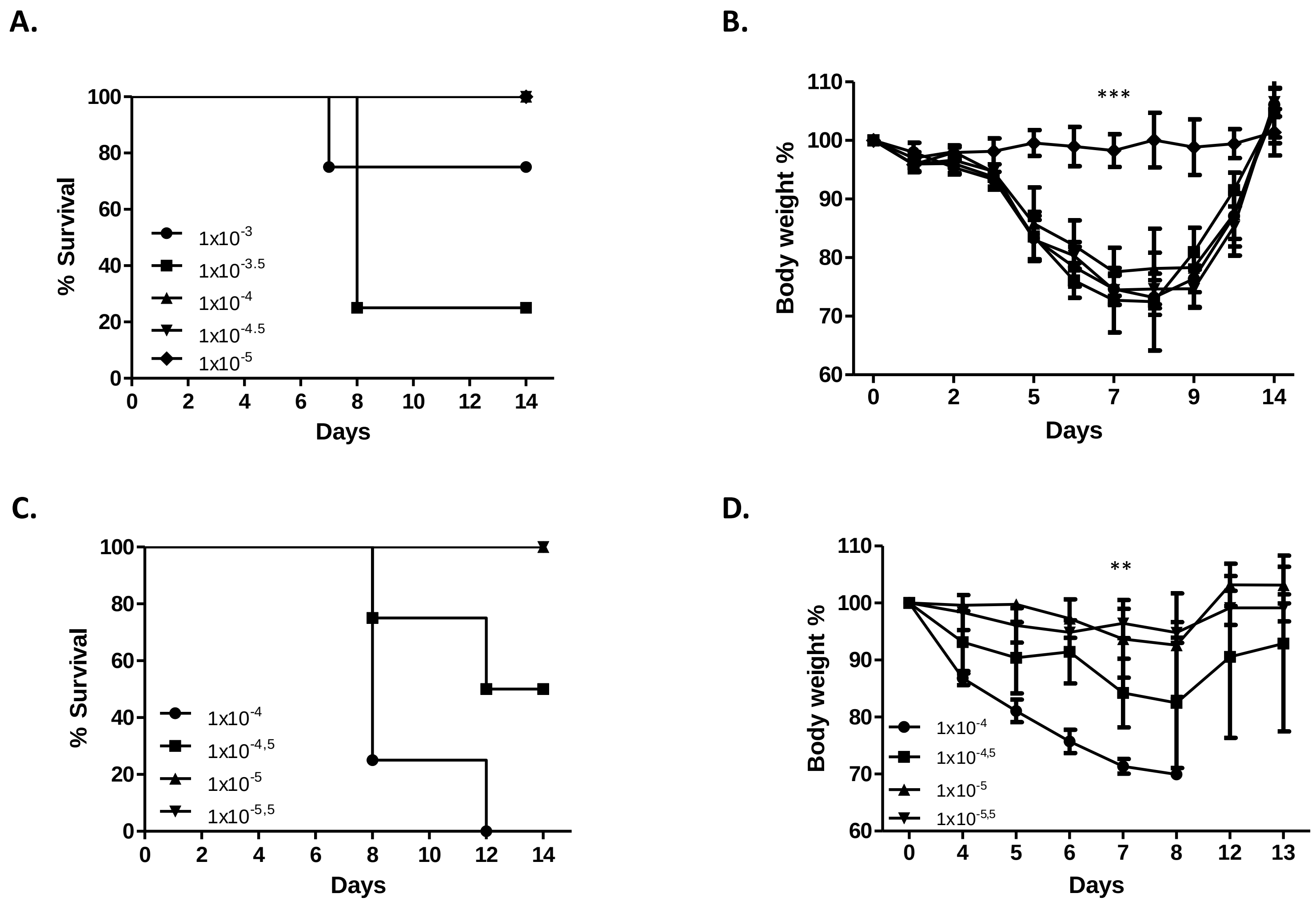

Supplement: S1 Table — (PDF) [file pone.0301664.s001.pdf]
