# Supplementary material for: Anti-hemagglutinin monomeric nanobody provides prophylactic immunity against H1 subtype influenza A viruses
Source: PLoS One. 2024 Jul 10;19(7):e0301664. doi: 10.1371/journal.pone.0301664 (PMC11236207; doi:10.1371/journal.pone.0301664)

S1 Fig. VHH protein analysis. Purified VHHs were visualized by Coomassie Blue staining after 15% SDS-PAGE.

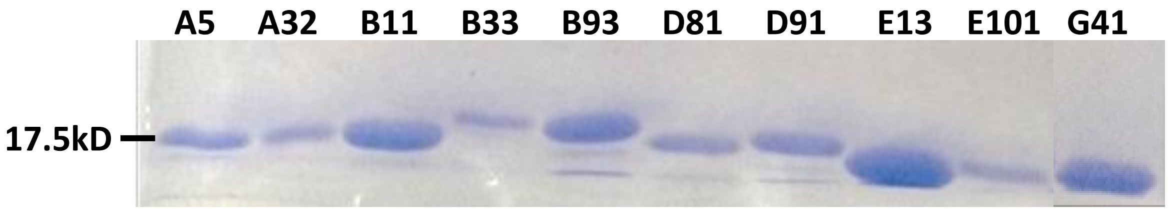

Supplement: S1 Fig — (PDF) [file pone.0301664.s002.pdf]
