# Supplementary material for: Anti-hemagglutinin monomeric nanobody provides prophylactic immunity against H1 subtype influenza A viruses
Source: PLoS One. 2024 Jul 10;19(7):e0301664. doi: 10.1371/journal.pone.0301664 (PMC11236207; doi:10.1371/journal.pone.0301664)

S2 Fig. VHH sequence analysis. A. Table depicting the V/J germline origin analysis. Protein nanobody sequences were compared with the Lama glama Ig set from the IMGT reference directory. Information about and percentage of identity, difference on residues composition on the CDR1 and CDR2, FRs and V-domains compared with the closest genes and alleles are shown. The accumulated mutations in V domains compared to the germline is indicated. B. Phylogenetic analysis for the ten VHHs under study. The percentage of similarity compared to the common ancestor is indicated on the right for each VHH clone.

A.

	*Allele (Identity %)		# aa changed		Total mutations in V-DOMAIN
VHH	VH-GENE	JH-GENE	CDR1-CDR2	FRs	
A5	IGHV3S2*01 (79.4%)	IGHJ4*01 (92.9%)	6	14	20
A32	IGHV3S1*01 (81.0%)	IGHJ5*01 (84.6%)	9	10	19
B11	IGHV3S1*01 (80.0%)	IGHJ5*01 (84.6%)	9	10	19
B33	IGHV3S1*01 (76.5%)	IGHJ2*01 (100%)	11	11	22
B93	IGHV3S2*01 (75.0%)	IGHJ4*01 (92.9%)	6	18	24
D81	IGHV3S2*01 (76.3%)	IGHJ4*01 (92.9%)	11	12	23
D91	IGHV3S1*01 (81.2%)	IGHJ4*01 (85.7%)	7	8	15
E13	IGHV3S3*01 (80.2%)	IGHJ2*01 (100%)	7	10	17
E101	IGHV3S2*01 (80.4%)	IGHJ3*01 (90.9%)	6	13	19
G41	IGHV3S3*01 (69.5%)	IGHJ4*01 (91.7%)	8	19	27

B.

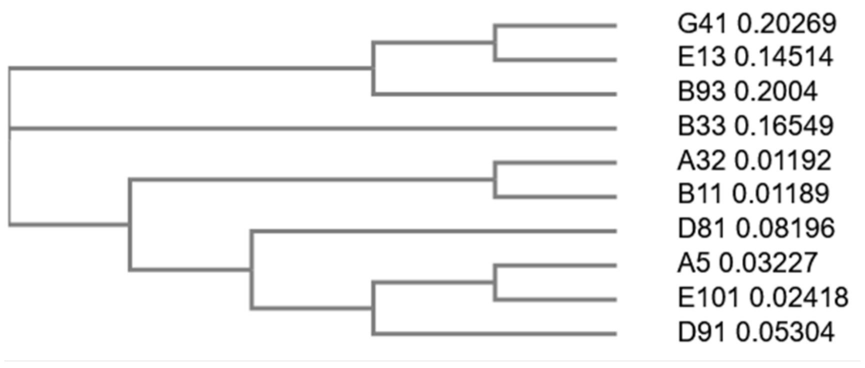

Supplement: S2 Fig — (PDF) [file pone.0301664.s003.pdf]
