# Supplementary material for: Anti-hemagglutinin monomeric nanobody provides prophylactic immunity against H1 subtype influenza A viruses
Source: PLoS One. 2024 Jul 10;19(7):e0301664. doi: 10.1371/journal.pone.0301664 (PMC11236207; doi:10.1371/journal.pone.0301664)

S3 Fig. VHH specific reactivity to HA0. In order to elucidate specific recognition of HA1 or HA2, treatment with acid buffer and DTT was performed on the viral antigen coated in plate. Nanobodies were added in a set of 5-fold serial dilutions A. E13, and B. B33 were tested for their capacity to bind HA in the context of the viral particle for hu/Arg/09, hu/Arg/09 ma, and hu/PR8/34. Controls of non-treated virus were run in parallel for each VHH. Absorbance obtained at 450nm is indicated for treated (T hu/Arg/09, T hu/Arg/09 ma, and T hu/PR8/34) and non-treated viruses (hu/Arg/09, hu/Arg/09 ma, and hu/PR8/34).

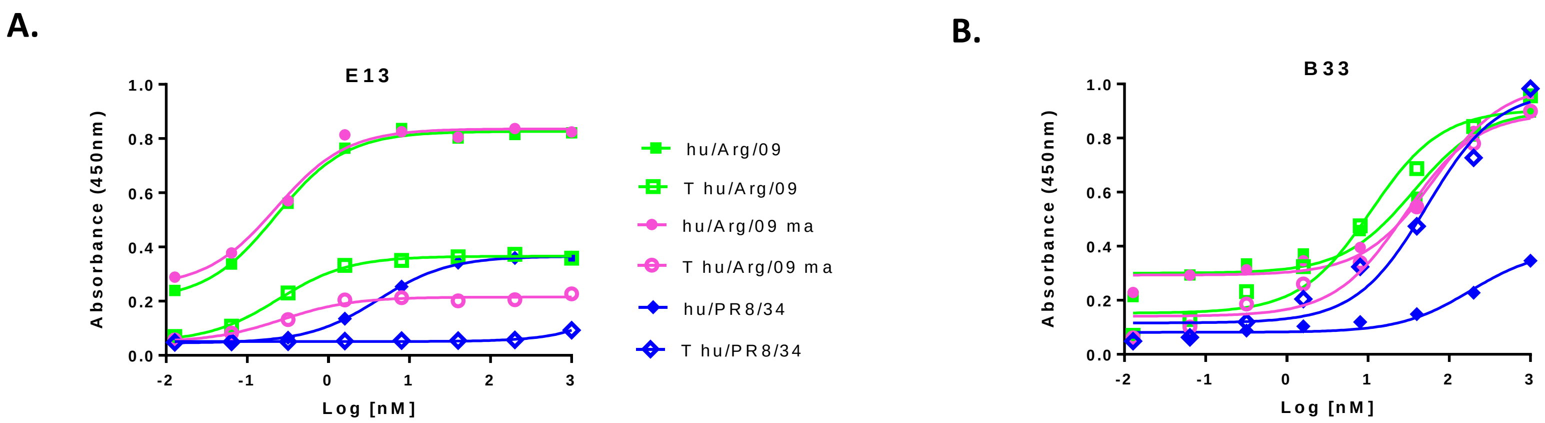

Supplement: S3 Fig — (PDF) [file pone.0301664.s004.pdf]
