# Supplementary material for: Anti-hemagglutinin monomeric nanobody provides prophylactic immunity against H1 subtype influenza A viruses
Source: PLoS One. 2024 Jul 10;19(7):e0301664. doi: 10.1371/journal.pone.0301664 (PMC11236207; doi:10.1371/journal.pone.0301664)

S4 Fig. E13 and G41 neutralizing activity against viruses of subtypes H1 and H3. A. VHHs E13 and G41 were challenged with H1N1 hu/Ca/09ma NLuc virus, or C, H3N2 ty/OH/04 NLuc, B. Immune serum against hu/Ca/09 was challenged with hu/Ca/ 09 ma NLuc or D, H3N2 ty/OH/04 NLuc, E. Phylogenetic analysis based on the HA sequence of viruses of subtypes H1, H3, H5, and H9. Phylogenetic group 1 represented by pandemic H1N1 viruses, from clade 1A3.3.2: hu/Ca/09, hu/Arg/09ma, hu/Arg/09, hu/Ca/09ma; from clade 1A.2 the post-pandemic virus sw/SD/18; from clade 1B.2 the pre-pandemic viruses hu/PR8/34 and hu/Bri/07; while the avian viruses, H1 rt/Del/09, and H9 gf/HK/99, H5 hu/Viet/04 have a common ancestor different from all the previous ones. Phylogenetic group 2 (subtype H3) represented by the post-pandemic virus eq/Arg/12, the pre-pandemic viruses ty/OH/04, and sw/Arg/08, and the contemporary to the pandemic event hu/Perth/09.

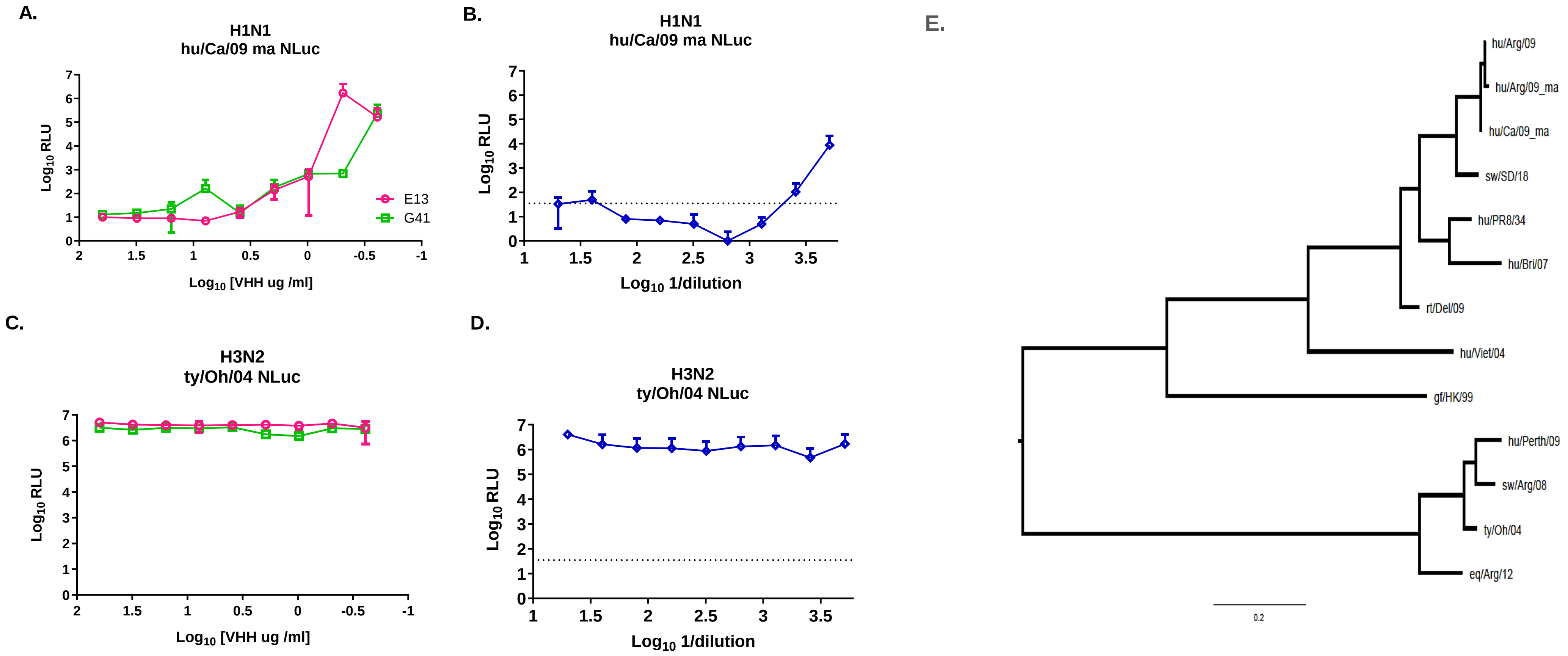

Supplement: S4 Fig — (PDF) [file pone.0301664.s005.pdf]
