# Supplementary material for: Anti-hemagglutinin monomeric nanobody provides prophylactic immunity against H1 subtype influenza A viruses
Source: PLoS One. 2024 Jul 10;19(7):e0301664. doi: 10.1371/journal.pone.0301664 (PMC11236207; doi:10.1371/journal.pone.0301664)

S5 Fig. Pathogenicity of hu/Arg/09 along mouse adaption process. A, Body weight. BALB/c mice were infected with wild-type virus and four days later, their lungs were dissected out to generate the first passage of mouse adapted virus. Subsequently, the lung lysates were used for intranasal infection of mice to generate Passage 2, which was subjected six additional mouse lung passages. B, Replication level for each passage of virus was determined as viral titers in the lungs. Data are expressed as mean ± SD of viral titers (Log_{10} $TCID_{50}$/ml) in the lungs of each group of mice (n=5) from three separate experiments. C. Replication kinetics on MDCK cell monolayers. To determine multistep growth curves of the virus in vitro, MDCK cells were infected in duplicate with MOI 0.05 PFU/cell of hu/Arg/09 or hu/Arg/09ma viruses, respectively. After 60 min absorption at 37°C, the cells were washed and cultured with serum-free DMEM containing TPCK-treated trypsin (2 µg/ml) and antibiotics for 0, 12, 24, 36, 48, 60, and 72 hours post infection. At each time point infected and a non-infected control were observed for cytopathic effect. Cell supernatant were centrifuged at 3000 x g for 10 min, and stored at -80°C until titration by $TCID_{50}$ assay. One-way ANOVA: A: Tukey Test. *: p<0,05; **: p<0,01; ***: p<0,005. A: Passage Body weight 3, 4, 5 vs 6 y 7; viral titer passage 5 vs 6.

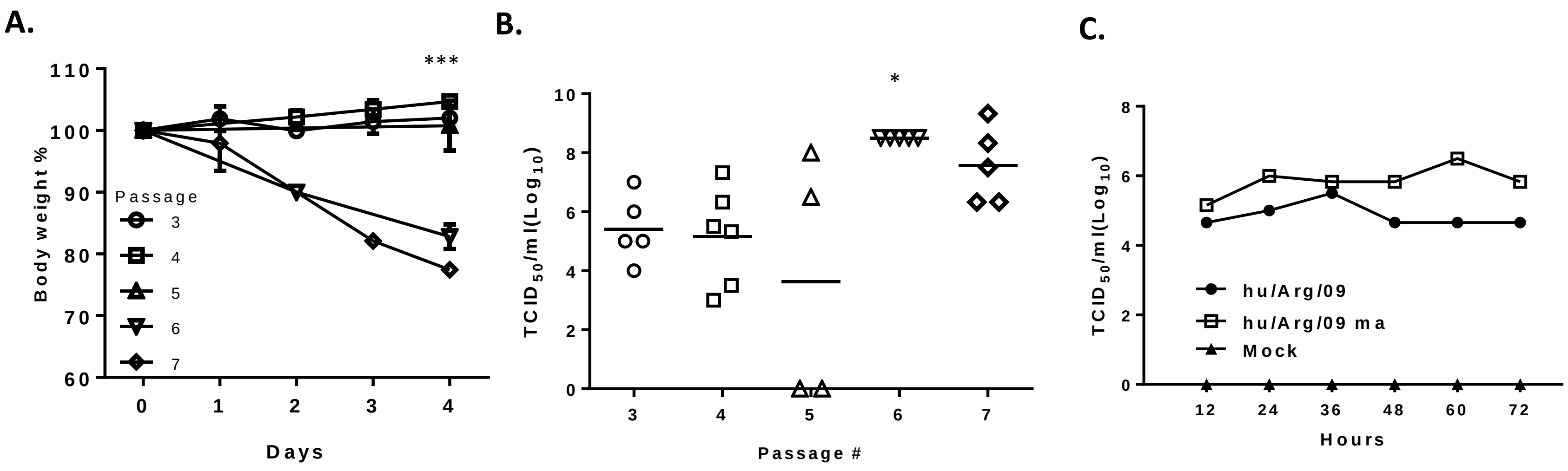

Supplement: S5 Fig — (PDF) [file pone.0301664.s006.pdf]
