# Supplementary material for: Anti-hemagglutinin monomeric nanobody provides prophylactic immunity against H1 subtype influenza A viruses
Source: PLoS One. 2024 Jul 10;19(7):e0301664. doi: 10.1371/journal.pone.0301664 (PMC11236207; doi:10.1371/journal.pone.0301664)

S6 Fig. Median Lethal Dose (LD_{50}) of viral strains used for challenge. Survival curve for A, hu/Arg/09ma and C, hu/PR8/34; Body weight curve for B, hu/Arg/09ma and D, hu/PR8/34. Female mice BALB/c were infected by intranasal route (50ul/dose) dilutions of virus as shown in the figure. Dilutions used for inoculated viral strains were: for hu/Arg/09ma, $1x10^{-3}$, $1x10^{-3.5}$, $1x10^{-4}$, $1x10^{-4.5}$, $1x10^{-5}$; and for huPR8/34 $1x10^{-4}$, $1x10^{-4.5}$, $1x10^{-5}$, $1x10^{-5.5}$. Survival and Body weight were monitored daily for 14 days after infection. Error bars represent standard deviations of the mean. One-way ANOVA, Tukey Test. *: p<0,05; **: p<0,01; ***: p<0,005. Survival curves, Mantel-Cox Test.

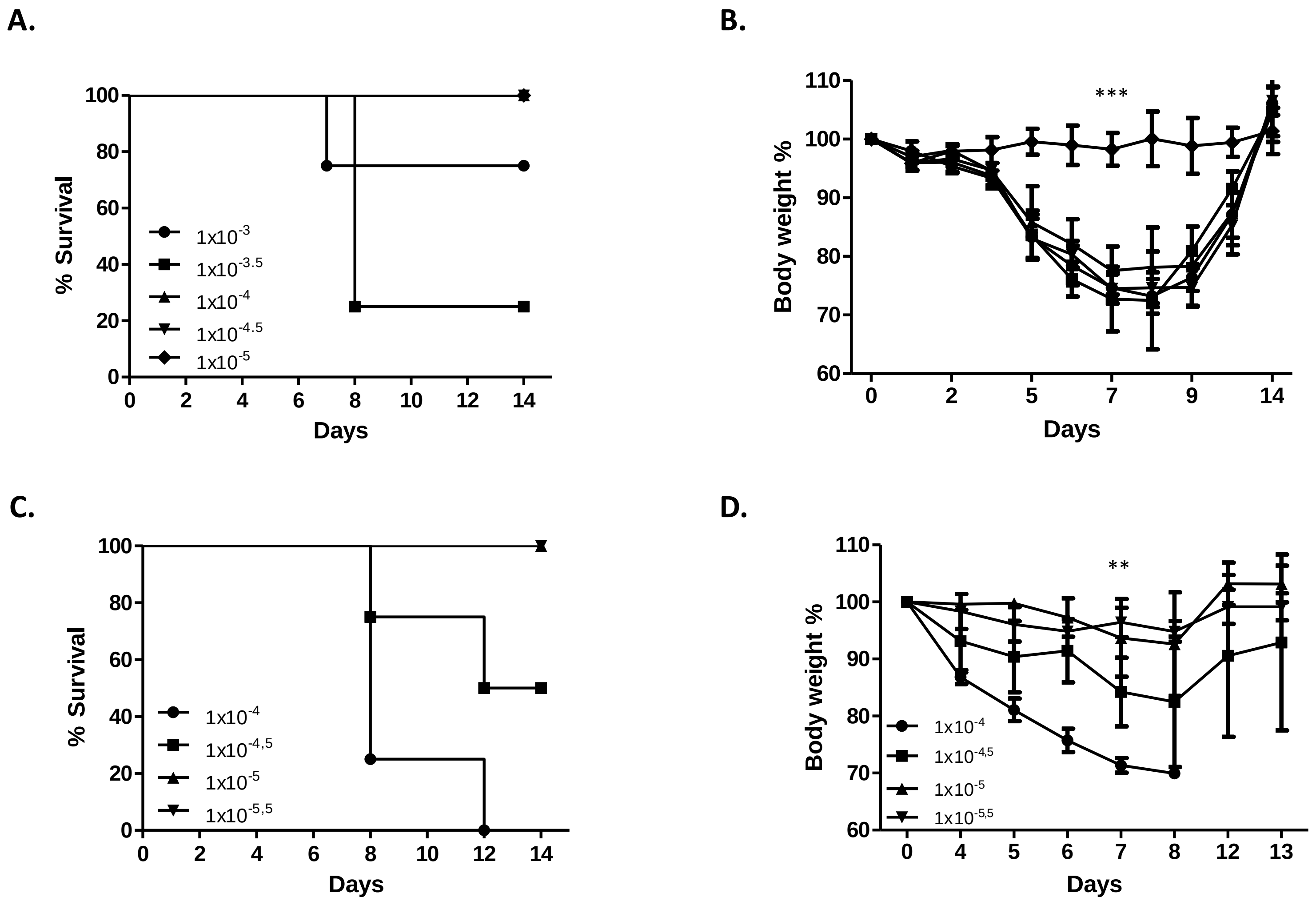

Supplement: S6 Fig — (PDF) [file pone.0301664.s007.pdf]
